# A translational protocol optimizes the isolation of plasma-derived extracellular vesicle proteomics

**DOI:** 10.1038/s41598-025-08366-8

**Published:** 2025-07-07

**Authors:** Jussara Ríos de los Ríos Reséndiz, Freya Herrmann-Sim, Liliana Wilkesmann, Dominic Helm, Martin Schneider, Giorgia Campione, Klara Plügge, Giovanni Greiner, Leonie Lazaro García, Julia Berker, Karsten Richter, Lin Zielske, Wolf-Karsten Hofmann, Katharina Clemm von Hohenberg

**Affiliations:** 1 Junior Clinical Cooperation Unit Translational Lymphoma Research, German Cancer Research Center (DKFZ) Heidelberg, 68169 Mannheim, Germany; 2 Department of Hematology and Oncology, Medical Faculty Mannheim, University Heidelberg, 68167 Mannheim, Germany; 3 Proteomics Core Facility, German Cancer Research Center (DKFZ), 69120 Heidelberg, Heidelberg, Germany; 4 Karlsruhe Institute of Technology, 76131 Karlsruhe, Germany; 5 Electron Microscopy Core Facility, German Cancer Research Center (DKFZ), 69120 Heidelberg, Heidelberg, Germany

**Keywords:** Extracellular vesicles, Liquid biopsy, Plasma, Translation, Proteomics, Pre-analytics, Cancer, Cell biology, Biomarkers, Medical research, Molecular medicine, Oncology, Biotechnology, Proteomics

## Abstract

In translational research and clinical routine, liquid biopsy is a promising tool to direct individually targeted treatments. Among the components of liquid biopsy, extracellular vesicles (EVs) carry manyfold molecular cargo and are increasingly being studied for biomarker identification. In order to identify potential confounding factors and determine optimal conditions when studying blood-derived EV proteins, the impact of pre-analytical variables needs to be assessed. Here we establish an EV enrichment for proteomic analysis workflow in a real-world clinical setting in which we evaluate variables from blood collection through protein preparation and storage for mass spectrometry (MS). We assess hemolysis, particle concentration and size, protein quantity, protein markers and comprehensive proteomic analysis using mass spectrometry to assess the influence of different pre-analytical variables like blood collection tubes, transportation of blood samples and delayed processing. Under these conditions, density gradient and size exclusion chromatography using Sepharose CL-4B show good EV enrichment. For MS, lysis with increased protease inhibitors shows high protein yields while TCA protein precipitation results in high numbers of identified proteins. In summary, we develop here an optimized protocol for the analysis of plasma EV-derived proteomics, evaluating pre-analytical variables relevant for implementation in a clinical setting.

**Supplementary Information:**

The online version contains supplementary material available at 10.1038/s41598-025-08366-8.

## Introduction

 Liquid biopsy is increasingly used in personalized medicine to tailor cancer treatments by analyzing cell-free DNA (cfDNA), circulating tumor DNA, and microRNA. While these biomarkers have entered routine clinical use^1^ their predictive power is limited, and proteomic analysis is needed for functional tumor insights. Proteomic data, however, is more challenging to obtain due to the instability of proteins and the inability to amplify them like nucleic acids, making it one of the main hurdles in liquid biopsy applications^2^.

Extracellular vesicles (EVs), small cell-derived particles carrying proteins, lipids, metabolites, and nucleic acids, have emerged as a promising source of tumor-specific biomarkers. These vesicles, found in all body fluids^3^ can provide valuable insights into tumor biology^4,5^ and serve as drug delivery vehicles due to their low immunogenicity and ability to target cells^6^. Over the last two decades, EVs have shown great potential for clinical applications, but translating EV research into practical use has faced challenges^7,8^, particularly regarding standardization, reproducibility^9^ and sample handling.

The quality of biofluids, especially blood, is critical for EV analysis^10^. Pre-analytical factors such as collection, transportation, storage, and processing affect EV quality and diagnostic outcomes^11,12^. Transportation of blood samples to the laboratory introduces mechanical stress that can compromise EV integrity^13^. Furthermore, red blood cells undergo ex vivo hemolysis when kept at 40 °C or above^14^ while refrigeration during transportation may preserve EVs, but is energy-intensive and not always effective^15^. Therefore, understanding the impact of transportation conditions, including temperature, on EV quality is essential for reliable results.

After blood collection and transport, EVs must be isolated from plasma. Techniques like differential or density gradient ultracentrifugation and size exclusion chromatography are commonly used^16,17^ but preserving EVs free from plasma protein contamination remains a challenge. Studies have shown that pre-analytical and analytical variables can significantly affect the outcomes of EV proteomic analysis, limiting its diagnostic power^18–21^. Given that many clinical settings face logistical challenges, such as patient care sites being distant from research laboratories^22^ optimizing transport and processing methods is key for successful clinical translation.

Storage of isolated EVs is another critical step. While recent evidence suggests that storing EVs in proteinaceous buffers may preserve their properties^23–27^ protocols for long-term storage and transportation are still being refined. In this study, we test different storage conditions and isolation methods for EVs in a clinical research setting, aiming to optimize the entire workflow from blood collection to mass spectrometry analysis. Our goal is to make these protocols scalable and applicable in broader clinical contexts, enhancing the utility of EVs in translational research and personalized cancer treatment.

This work investigates real-world challenges in EV proteomic analysis, exploring how to implement laboratory-optimized protocols in a clinical research environment. By improving methods for handling and analyzing EVs, we hope to advance their clinical application, ultimately benefiting patient care and personalized treatment strategies.

## Results

**Fig. 1:**
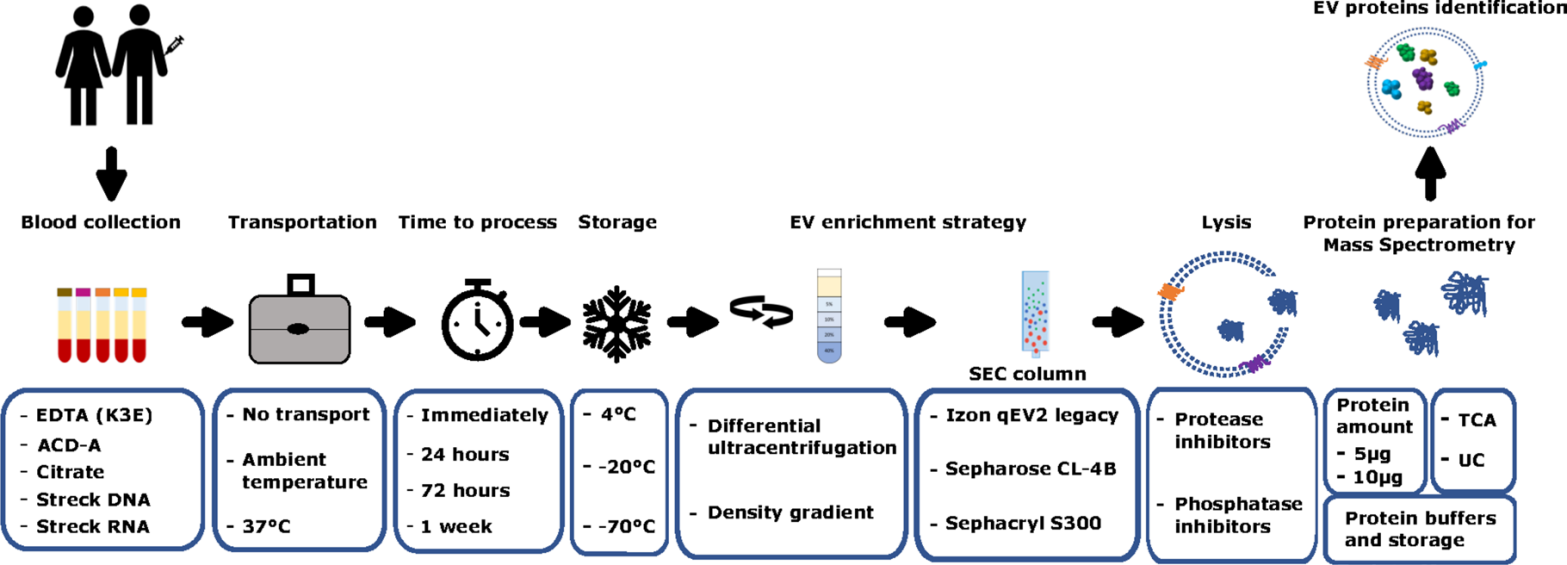
Overview of pre-analytical variables optimized in a clinical-translational setting.

In order to enhance applicability of proteomic extracellular vesicle (EV) analysis in a clinical and translational setting we set out to comprehensively test and optimize several preanalytical and procedural factors (Fig. 1).

### Blood collection tubes

**Fig. 2:**
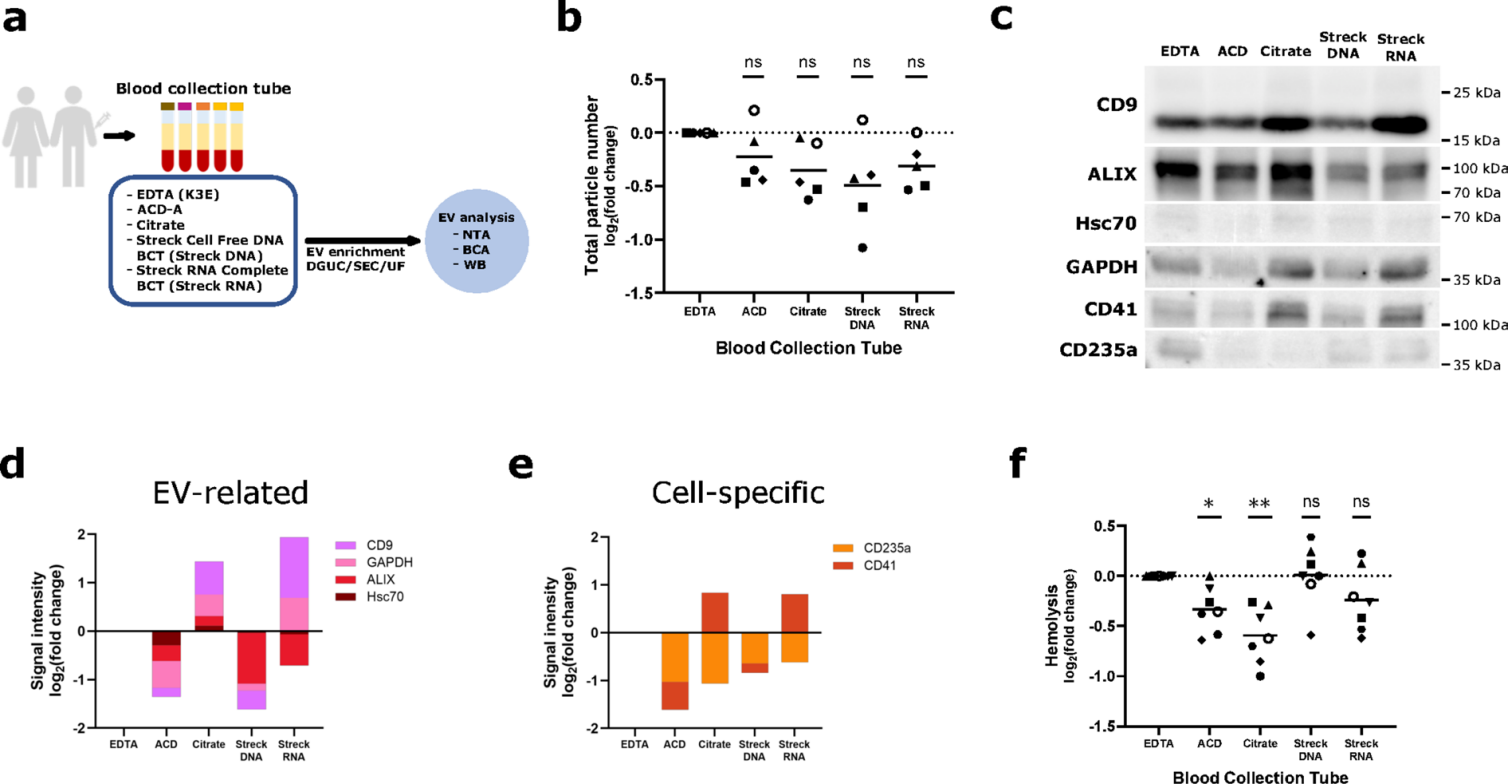
Blood collection tubes (BCTs) impact hemolysis and characteristics of recovered nanoparticles. (**a**) Peripheral blood was collected in five different BCTs each time. EV enrichment was performed by density gradient ultracentrifugation (DGUC), followed by size exclusion chromatography (SEC) and then ultrafiltration (UF). EV-enriched fractions were analyzed by nanoparticle tracking analysis (NTA), bicinchoninic acid assay (BCA) (see Supplementary Fig. S1 online) and western blot (WB). (**b**) Particle numbers obtained after DGUC and SEC, measured by NTA. Data normalized to the reference BCT (EDTA). Data derived from *n*=5 biological replicates. Mean of normalized value is displayed for each BCT and statistical significance is assessed using one sample t test (Bonferroni-Holm-adjusted p-values) with *p*=0.1545 (ACD), *p*=0.1384 (Citrate), *p*=0. 1384 (StreckDNA) and *p*=0. 1384 (StreckRNA). (**c**) Protein lysates from particles purified from each BCT analyzed by Western Blot. Representative immunoblotting image to characterize particles obtained according to EV- (CD9, ALIX, Hsc70, GAPDH) and cell-specific (CD41, CD235a) markers. Original blots are presented in Supplementary Fig. S8 online. (**d-e**) Band intensity quantification of representative WB in (**c**). (**f**) Hemolysis measurement of platelet-poor plasma obtained from the BCTs tested, measured by absorbance at 414 nm. Data normalized to the reference BCT (EDTA). Data derived from *n*=7 biological replicates. Mean of normalized value is displayed for each BCT and statistical significance is assessed using one sample t test (Bonferroni-Holm-adjusted p-values) with *p=0.0252 (ACD), **p=0.0056 (Citrate), *p*=0.9338 (StreckDNA) and *p*=0.1846 (StreckRNA). In (**b**) and (**f**) single symbols signify independent biological replicates.

 In a first step, we compared different blood collection tubes (BCTs) currently used for liquid biopsy and for conventional routine blood tests in the clinic (Fig. 2a), namely EDTA, Citrate, ACD, Streck DNA and Streck RNA. Supplementary Table S1 online contains an overview of the cost of each of these preservation tubes. According to Nanoparticle Tracking Analysis (NTA), particle numbers isolated from ACD, Citrate, Streck RNA and Streck DNA tubes tend to be smaller compared to the conventional EDTA tubes (Fig. 2b). These results correspond to the amount of EV-derived protein from the different BCTs (see Supplementary Fig. S1 online). Only in Streck RNA tubes the protein yield is clearly reduced (see Supplementary Fig. S1 online). Vesicle size, on the other hand, is unaffected by different preservation anticoagulants (see Supplementary Fig. S1 online). EVs can derive from physiological sources or from blood cells ex vivo. Eventually, both of these fractions contribute to the total number of EVs isolated and quantified. Therefore, we further characterized EVs purified from the different BCTs. In contrast to the NTA quantification, EV-derived protein from Citrate and Streck RNA tubes contains more EV-associated markers^9^ than from EDTA (Fig. 2c, d, see Supplementary Fig. S1 online). At the same time, the red blood cell marker CD235a is clearly decreased in all non-EDTA tubes, suggesting fewer extra-physiological activation or fragmentation of red blood cells. ACD tubes show the lowest signal for CD41, potentially indicative of less platelet-derived EV release (Fig. 2c, e). Finally, an important quality control is the measurement of hemolysis. We tested hemolysis using absorption at 414 nm in platelet-poor plasma from all tested BCTs. Application of ACD or Citrate instead of EDTA tubes substantially reduces hemolysis while other BCTs do not meaningfully improve this aspect (Fig. 2f).

In summary, based on enrichment of EV-associated protein markers and on reduction of hemolysis, for studies of EV-derived proteomics we recommend blood draw into Citrate tubes. Alternatively, despite showing lower EV marker signals, ACD-derived EVs are accompanied by reduced CD235a, CD41 and a trend towards lower hemolysis than EDTA. Therefore, considering the great concern about platelet/red blood cell (RBC)-derived EVs, ACD tubes might be further considered for blood-derived proteomic EV studies.

## Transportation temperature

**Fig. 3:**
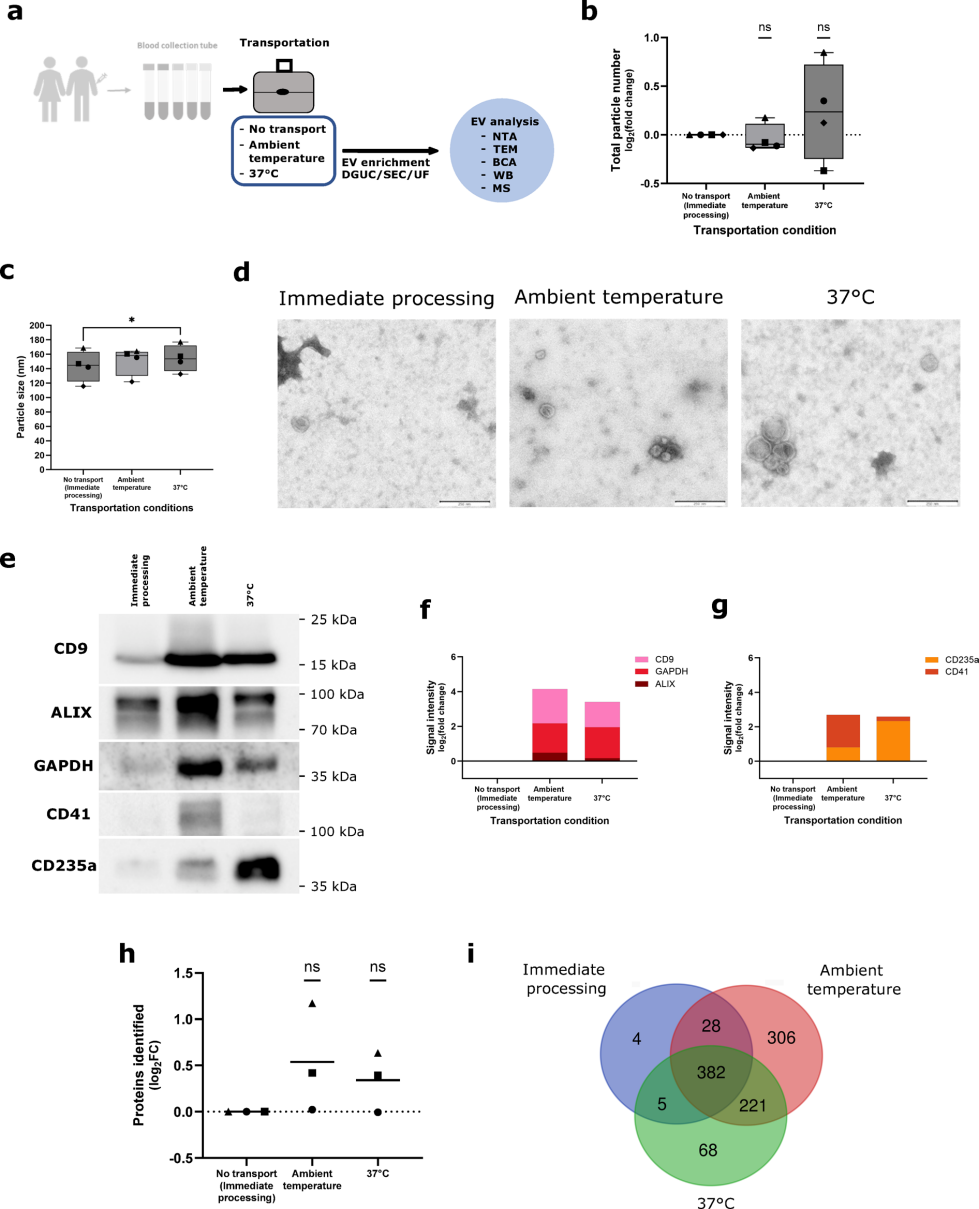
Transportation temperature increases the variance of particles recovered and differentially affects blood cell types. (**a**) A set of three peripheral blood samples was each collected in *n*=4 biological replicates in a Streck Cell-Free DNA BCT. For the no transport condition, platelet-poor plasma (PPP) was prepared immediately after blood draw. For the transport conditions, full blood was sent at ambient temperature or in a container at 37 °C. EV enrichment was performed by DGUC, followed by SEC and then UF. EV-enriched fractions were analyzed by nanoparticle tracking analysis (NTA), transmission electron microscopy (TEM), bicinchoninic acid assay (BCA) (see Supplementary Fig. S2 online), western blot (WB) and mass spectrometry (MS). (**b**) Particle numbers obtained after DGUC and SEC, measured by NTA. Data normalized to the control condition of immediately processed blood. Mean of normalized value is displayed for each transport condition and statistical significance is assessed using one sample t test with *p*=0.6328 (Ambient temperature) and *p*=0.4156 (37 °C). (**c**) Median particle size measured by NTA. In (**b**) and (**c**) symbols signify independent biological replicates. Mean particle size is displayed for each transport condition and statistical significance is assessed using paired t test (Bonferroni-Holm-adjusted p-values) with *p*=0.3878 (Ambient temperature vs. no transport), *p=0.0441 (37 °C vs. no transport) and *p*=0.4976 (37 °C vs. ambient temperature). (**d**) Representative images taken by transmission electron microscopy (TEM) of particles purified from each condition. Scale bar = 250 nm. (**e**)-(**g**) Protein lysates from particles purified from each transportation condition analyzed by Western Blot. (**e**) Representative immunoblotting image to characterize particles obtained according to EV and cell-specific markers. Original blots are presented in Supplementary Fig. S9 online. (**f**), (**g**) Mean of WB signal quantification of individual samples (*n*=2 biological replicates). (**h**) Number of proteins identified by mass spectrometry in each condition from three biological replicates each. Mean normalized to the no transport condition is displayed and statistical significance is assessed using one sample t test with *p*=0.2511 (ambient temperature) and *p*=0.2092 (37 °C). (**i**) Venn diagram comparing the proteins present in the three replicates.

We tested the impact of different transportation temperatures and modalities on EV characteristics (Fig. 3). In the standard condition, full blood was centrifuged to produce platelet-poor plasma (PPP, see Methods section for detailed protocol) immediately after blood draw, and then transferred to the processing laboratory facility (Fig. 3a). In a second condition, full blood was transported to the laboratory at ambient temperature, with outside temperatures between 5 °C and 15 °C (see Supplementary Fig. S2 online). All further processing took place in the laboratory facility. The third experimental condition was identical to the second, with the exception that the full blood was strictly kept at 37 °C in a specialized transportation box. As far as particle numbers and EV-derived protein amounts are concerned, transport at 37 °C induces an increase in variability (Fig. 3b, see Supplementary Fig. S2 online). Variability in hemolysis is greater at both transportation conditions (see Supplementary Fig. S2 online). At the same time, size of EVs increases when transporting the full blood at 37 °C (Fig. 3c-d). Heat-induced extraphysiological changes of EV number and composition are confirmed by NTA (Fig. 3b, c) and by transmission electron microscopy (Fig. 3d). Next, we tested the exact protein composition of the purified vesicles in the three conditions. Interestingly, we were able to identify a drastic increase in vesicle-derived markers in conditions of full blood transport (Fig. 3e, f), Western Blot analysis and hemolysis measurement confirms ex vivo EV secretion of platelet or red blood cell origin in conditions of full blood transport (Fig. 3e-g, see Supplementary Fig. S2 online). Of note, CD235a exhibits a more pronounced increase in the condition at 37 °C while CD41 signal shows a trend to be higher at ambient temperature, suggesting that cell types might be differentially affected by each.

condition. In biomarker studies, translational researchers are interested in physiological rather than artificial EV content. Therefore, we characterized in more detail the ex vivo-added protein content by mass spectrometry (Fig. 3h-i). Here, we show that 527 proteins in the ambient temperature condition, and 289 proteins in the 37 °C condition are exclusively identified in transported samples and therefore can be considered contaminants of the analysis (Fig. 3i). Transport at 37 °C leads to increased particle numbers, while the range of proteins identified from samples transported at ambient temperature more clearly exceeds the control condition. According to the functional enrichment analysis performed with Metascape (see Supplementary Fig. S2 online), platelet activation is, at least partially, responsible for this increase in identified peptides. However, despite the differences in protein numbers, principal component analysis shows clustering of biological replicates and only limited influence of transportation condition on total sample composition (see Supplementary Fig. S2 online).

In summary, peripheral blood samples for EV-derived proteomic analysis should be either processed immediately to produce platelet-poor plasma in close proximity to the site of patient care or transported at room temperature. Transportation conditions are key to fine-tuning results of proteomic EV analysis.

## Needle-to-processing time

**Fig. 4:**
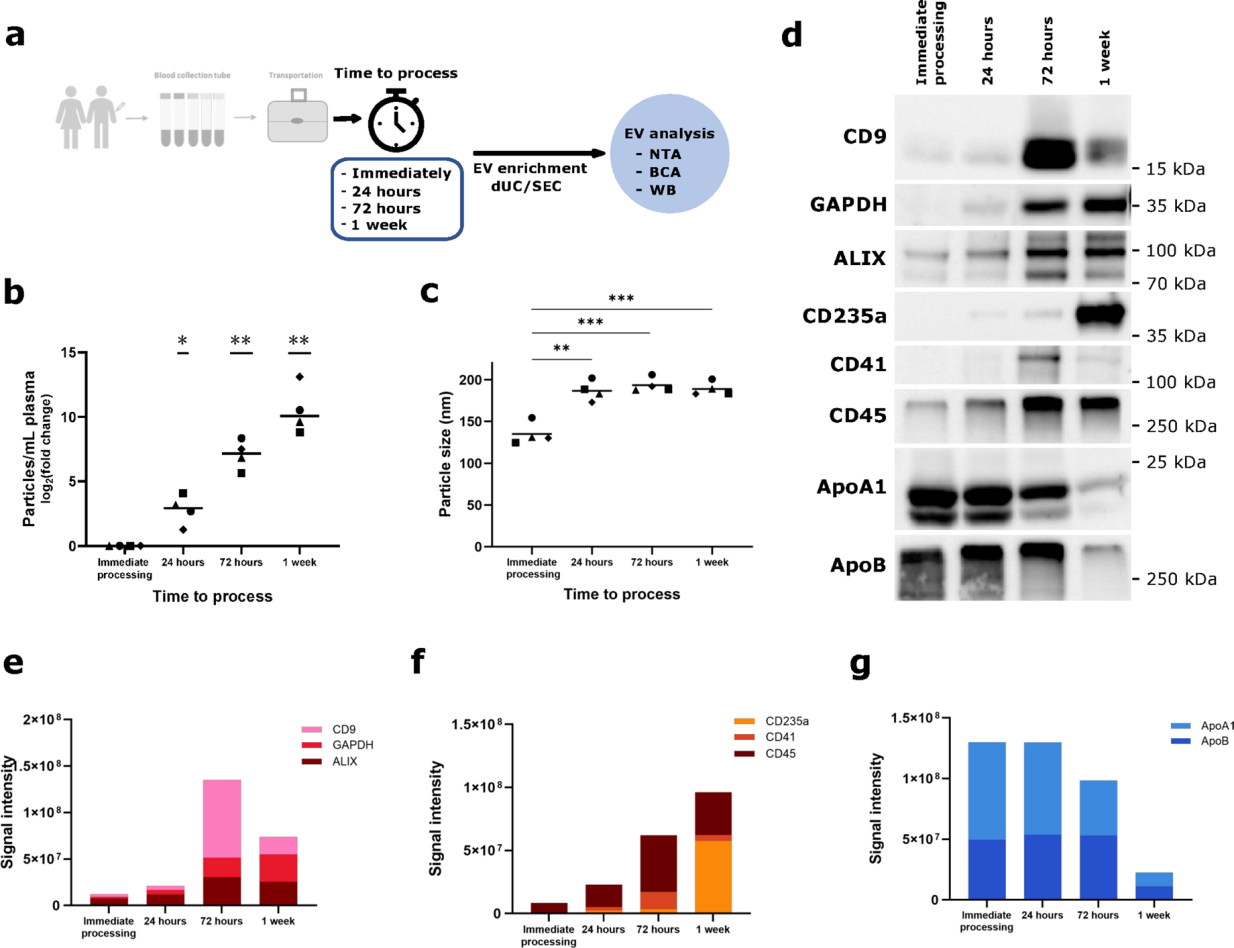
Particle number increases proportionally with needle-to-processing time. (**a**) A set of four peripheral blood samples was each collected in *n*=4 biological replicates Streck Cell-Free DNA BCT. EV enrichment was performed by differential ultracentrifugation (dUC), followed by SEC. EV-enriched fractions were analyzed by NTA, BCA and WB. (**b**) Particle numbers obtained from the different needle-to-processing times, measured by NTA. Data normalized to the control condition of immediately processed blood. Mean of normalized value is displayed for each needle-to-processing time and statistical significance is assessed using one sample t test (Bonferroni-Holm-adjusted p-values) with *p=0.0175 (24 h), **p=0.0033 (72 h) and **p=0.0033 (1 week). (**c**) Median size of particles recovered in EV-enriched fractions from the different needle-to-processing times. Mean value is displayed for each needle-to-processing time and statistical significance is assessed using paired t test (Bonferroni-Holm-adjusted p-values) with **p=0.0014 (24 h vs. immediate processing), ***p=0.0006 (72 h vs. immediate processing) and ***p=0.0006 (1 week vs. immediate processing). In (**b**) and (**c**) symbols signify independent biological replicates. (**d**) Representative immunoblotting image to characterize particles obtained according to EV markers, cell-specific markers and apolipoprotein markers. Original blots are presented in Supplementary Fig. S10 online. (**e**)-(**g**) Quantification of band intensities in (**d**).

 In real world clinical routine there are different set-ups for the processing of blood samples. Samples are either processed immediately (around the clock on-site laboratory service), within the first 24 h (e.g., 8 am to 5 pm on-site laboratory service), processed within 72 h (weekday on-site laboratory service, arrival on Friday night) or within one week (off-site laboratory service). We therefore decided to systematically test the influence of needle-to-processing time. We drew blood from healthy volunteers and processed it either immediately or kept it at room temperature for 24 h, 72 h or one week (Fig. 4a). We observed a dramatic increase in the amount of EVs and EV-derived protein when not processing full blood immediately and this increase is proportional to the needle-to-processing time (Fig. 4b, see Supplementary Fig. S3 online). Concurrently, we detect an increasing trend in hemolysis corresponding to the length of pre-processing time (see Supplementary Fig. S3 online). Interestingly, median vesicle size clearly increases as soon as samples are not processed immediately, likely reflecting a shift in the purified vesicle populations or vesicle fusion (Fig. 4c). Western Blot analysis confirms that additional vesicles and vesicle-derived proteins are predominantly from red blood cells (CD235), platelets (CD41) and leukocytes (CD45), thereby diluting physiological EV- and non-EV-derived proteins (Fig. 4d-g).

Based on these results, we strongly recommend to minimize needle-to-processing time for proteomic EV analysis.

## Storage temperature

The purification of EV-derived proteins requires a sophisticated multistep protocol. Even when processing patient samples with as little delay as possible (Fig. 4), the full purification process cannot be completed on the same day. Therefore, it must be the aim of a clinically actionable protocol to remove cellular content, the source of potentially contaminating EVs, as quickly as possible and then store platelet-poor plasma while maintaining EV quantity and quality. We proceeded to test the effect of different temperatures of PPP two weeks’ storage on the subsequently isolated extracellular vesicles (see Supplementary Fig. S4 online). While there seemed to be a tendency towards higher maintenance of vesicle numbers in −20 °C and − 70 °C as opposed to storage at 4 °C, neither vesicular Western Blot markers nor total protein cargo or particle size varied significantly between different storage temperatures (see Supplementary Fig. S4 online).

We conclude that, within a two weeks’ timeframe, storage temperature of PPP does not affect the quantity or quality of purified EV-derived proteins. However, storage at −20 °C or −70 °C might be preferred to 4 °C because of slightly better maintenance of EV quantity.

## Purification method

**Fig. 5:**
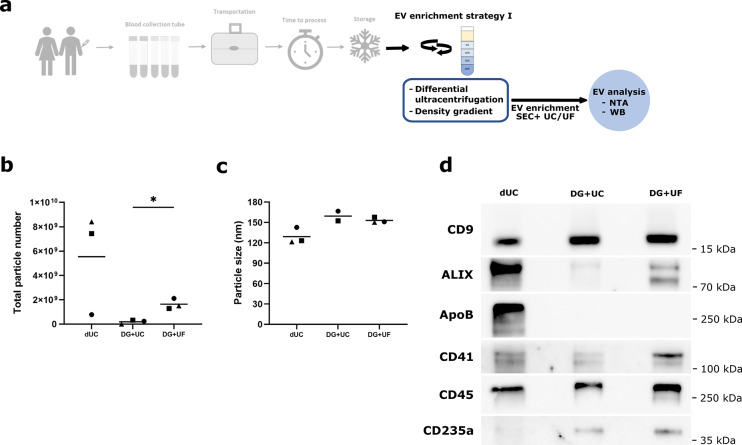
Density gradient ultracentrifugation combined with size exclusion chromatography and ultrafiltration recovers high particle numbers and reduces co-isolates. (**a**) A set of three peripheral blood samples was each collected in *n*=3 biological replicates in Streck Cell-Free DNA BCT. EVs were isolated using either differential ultracentrifugation (dUC) or density gradient ultracentrifugation (DG), followed by SEC. Following SEC, DG-purified EVs were concentrated either by ultracentrifugation (DG+UC) or by ultrafiltration (DG+UF). EV-enriched fractions were analyzed by nanoparticle tracking analysis (NTA) and western blot (WB). (**b**) Total particle recovery and (**c**) median particle size measured by NTA in EV-enriched fractions using different combinations of purification methods. In (**b**) and (**c**) mean values are displayed for each processing condition, and in (**b**) statistical significance is assessed using paired t test (DG+UC vs. DG+UF) with *p=0.0311. In (**b**) and (**c**) symbols signify independent biological replicates. (**d**) Representative immunoblotting of EV-enriched fractions obtained with the different strategies. Original blots are presented in Supplementary Fig. S11 online.

The actual isolation method used is commonly the main obstacle for clinical-translational units to encounter EV-derived analyses. This is due to logistical, technical and economic challenges on the one hand, and a lack of detailed and easily applicable protocols accompanied by reliable validation methods on the other hand. Consequently, we compared EV purification using differential ultracentrifugation (dUC) and size exclusion chromatography (SEC) to the isolation method of density gradient centrifugation (DGUC) and SEC, followed either by ultrafiltration or by another ultracentrifugation step to concentrate the sample (Fig. 5a). We observe a dramatic reduction in particles quantified by NTA in the DGUC samples as opposed to dUC (Fig. 5b), either due to particle loss during DGUC or due to false-positive detection of larger protein aggregates or apolipoproteins in the NTA analysis of dUC-treated samples. Furthermore, ultrafiltration recovers markedly more EVs from SEC eluate than another ultracentrifugation step (Fig. 5b). Disease-relevant markers are commonly found in small EVs^28^, we therefore included only the sEV fraction of dUC-processed samples in our analysis. Accordingly, dUC-isolated sEVs show a smaller median size than DGUC-isolated full vesicle populations (Fig. 5c). We then investigated the composition of dUC (+ SEC) vs. DGUC (+ SEC + UF/UC)-derived proteins. Strikingly, there is a massive reduction in co-purified apolipoproteins and platelet-derived vesicles when performing DGUC (Fig. 5d, see Supplementary Fig. S5 online, ApoB and CD41), accompanied by a slight increase in red blood cell-derived vesicles (Fig. 5d, CD235). The amount of CD9+ EVs recovered from ultracentrifugation following DGUC and SEC is comparable to ultrafiltration. However, ALIX+ and GAPDH+ EVs are much reduced with ultracentrifugation (Fig. 5 d, see Supplementary Fig. S5 online), implying that ultracentrifugation can only partially collect and concentrate the purified vesicles. Experimental costs for both methodologies are similar (see Supplementary Table S2 online). 

In summary, we recommend application of overnight DGUC as a first step of EV purification since it significantly reduces lipoprotein contamination of the obtained proteins. Following DGUC and SEC, ultrafiltration should be used to concentrate purified EVs and recover a broad spectrum of EV populations.

### Size exclusion chromatography (SEC) columns

**Fig. 6:**
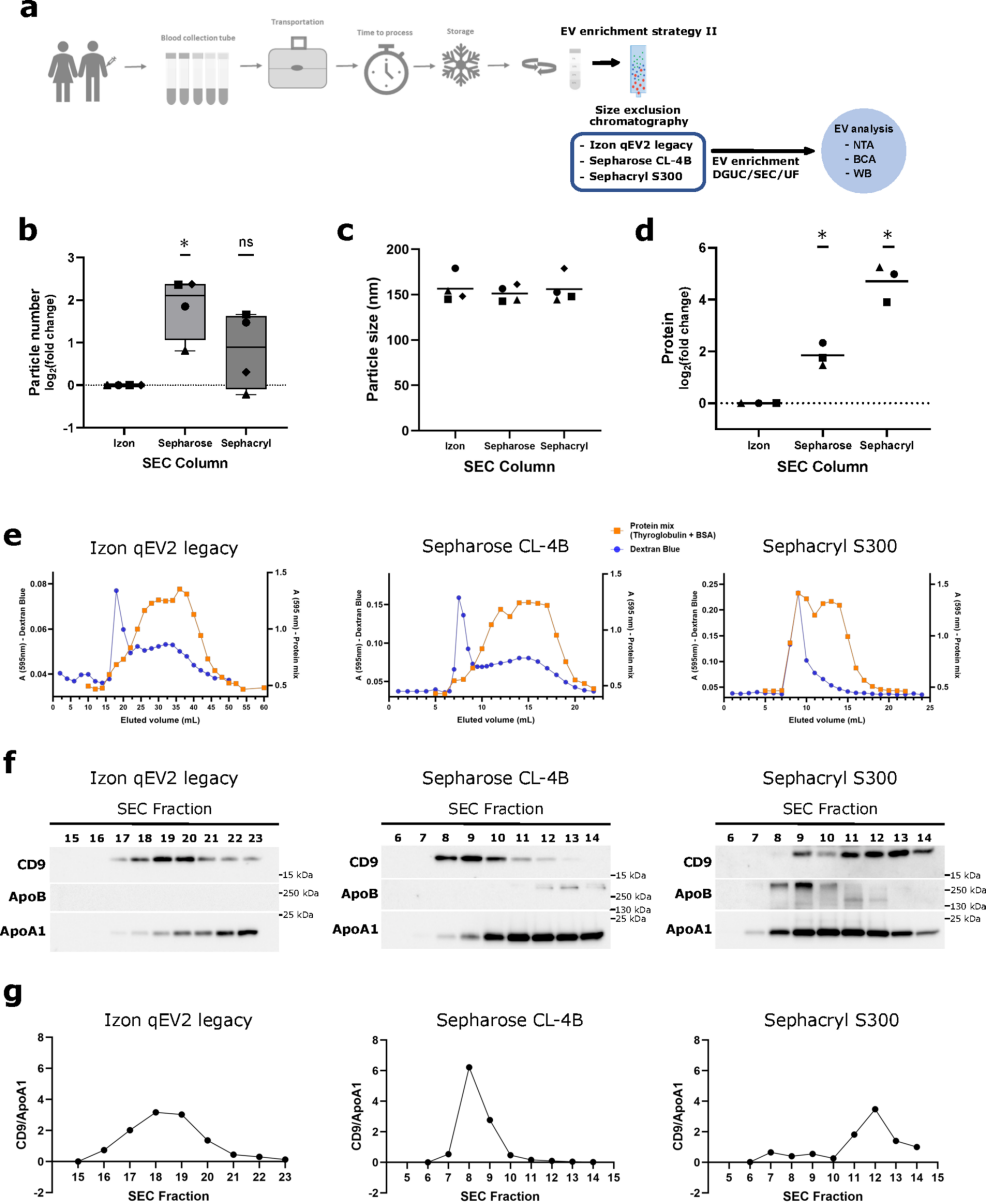
Size exclusion chromatography using Sepharose CL-4B shows high EV enrichment. (**a**) A set of three peripheral blood samples was each collected in *n*=4 biological replicates in Streck Cell-Free DNA BCT. In all cases, platelet-poor plasma (PPP) was first processed by density gradient, followed by SEC. After SEC, the EV-enriched fractions were concentrated by ultrafiltration. EV-enriched fractions were analyzed by nanoparticle tracking analysis (NTA), bicinchoninic acid assay (BCA) and western blot (WB). (**b**) Total particle recovery, (**c**) median particle size and (**d**) total amount of protein in EV-enriched fractions after size exclusion chromatography using different SEC resins, measured by (**b**), (**c**) NTA or (**d**) BCA. In (**b**)-(**d**) symbols signify independent biological replicates. In (**b**) and (**d**) mean of values normalized to Izon columns, is displayed, in (**c**) mean values are displayed for each SEC column. In (**b**) statistical significance is assessed using one sample t test (Bonferroni-Holm-adjusted p-values) with *p=0.0302 (Izon vs. Sepharose) and *p*=0.1745 (Izon vs. Sephacryl). In (**d**) statistical significance is assessed using one sample t test with *p=0.0182 (Izon vs. Sepharose) and *p=0.0152 (Izon vs. Sephacryl). (**e**) Representative images of standards run through each column to pre-assess their elution profiles. Dextran Blue (2 g/L), high molecular weight polymer, was used to predict the void volume and locate the EV-enriched peak. Thyroglobulin (2 g/L) and bovine serum albumin (BSA, 4 g/L), were used as representatives of a high and low molecular weight proteins, respectively. The signal corresponding to dextran blue was measured by absorbance at 595 nm. For protein quantification Bradford reagent was used and absorbance was measured at 595 nm. (**f**) Representative immunoblotting of 1mL fractions collected from each size exclusion chromatography to asses elution profiles using plasma as starting material. CD9 was selected as EV marker, and ApoB and ApoA1, as lipoprotein markers. Original blots are presented in Supplementary Fig. S12 online. (**g**) CD9/ApoA1 ratio calculated based on immunobloting signal quantification (*n*=2 independent biological replicates). Mean values are displayed.

 In addition to separation by density, separation by size using SEC is inevitable to obtain enriched EV populations and separation from lipoproteins of similar density. However, commercially available SEC columns are expensive and recommended to be used only a few times. Not uncommonly, transition times between different commercial column generations are too short for the completion of experimental series. Additionally, while commercially available SEC columns are usually packed in plastic columns, home-made SEC can be performed in re-usable glass columns, a potentially sustainable alternative. We therefore evaluated performance of commercially available in comparison to two types of home-made SEC columns (Fig. 6a). Notably, particle yield of home-made Sepharose CL-4B columns is significantly higher than from commercial Izon qEV columns (Fig. 6b). Home-made Sephacryl S300 columns do not yield higher EV numbers but elute a higher amount of protein (Fig. 6b and d). There are no conceivable differences in particle size (Fig. 6c). We set out to test elution profiles of the different columns by using dextran blue, a high molecular weight polymer with size characteristics similar to EVs^29^ and a mix of two proteins of different sizes, thyroglobulin and BSA. Elution profiles are similar for Izon qEV and Sepharose CL-4B columns (6e, see Supplementary Fig. S6 online), but in Sephacryl S300 columns even smaller proteins elute together with dextran blue (Fig. 6e). This confirms the high co-elution of proteins with home-made Sephacryl S300 columns as observed in the bulk analysis (Fig. 6d). These results are underlined by analyses from plasma-derived EVs (Fig. 6f-g). Again, the separation between EVs and lipoproteins is comparable in Izon qEV and in home-made Sepharose CL-4B columns, while in Sephacryl S300 columns there is a significant overlap between EV- and lipoprotein-containing peak fractions (Fig. 6f). Sepharose CL-4B exhibit a particularly high enrichment index, consisting of a higher peak in a reduced number of fractions (Fig. 6g) and performance remains high after 7 and 11 cycles of usage (see Supplementary Fig. S6 online). At the same time, self-made Sepharose CL-4B columns are an economically and ecologically more sustainable option. Furthermore, our cost-effectiveness analysis (see Supplementary Table S2) shows lower total cost of Sepharose CL-4B in comparison to the commercially available column.

In summary, Sepharose CL-4B columns are a cost-effective and sustainable alternative to commercially available SEC columns and exhibit reliable high performance regarding the separation of EVs from protein aggregates and lipoproteins.

## Protein lysis and delivery to the mass spectrometry core facility

**Fig. 7:**
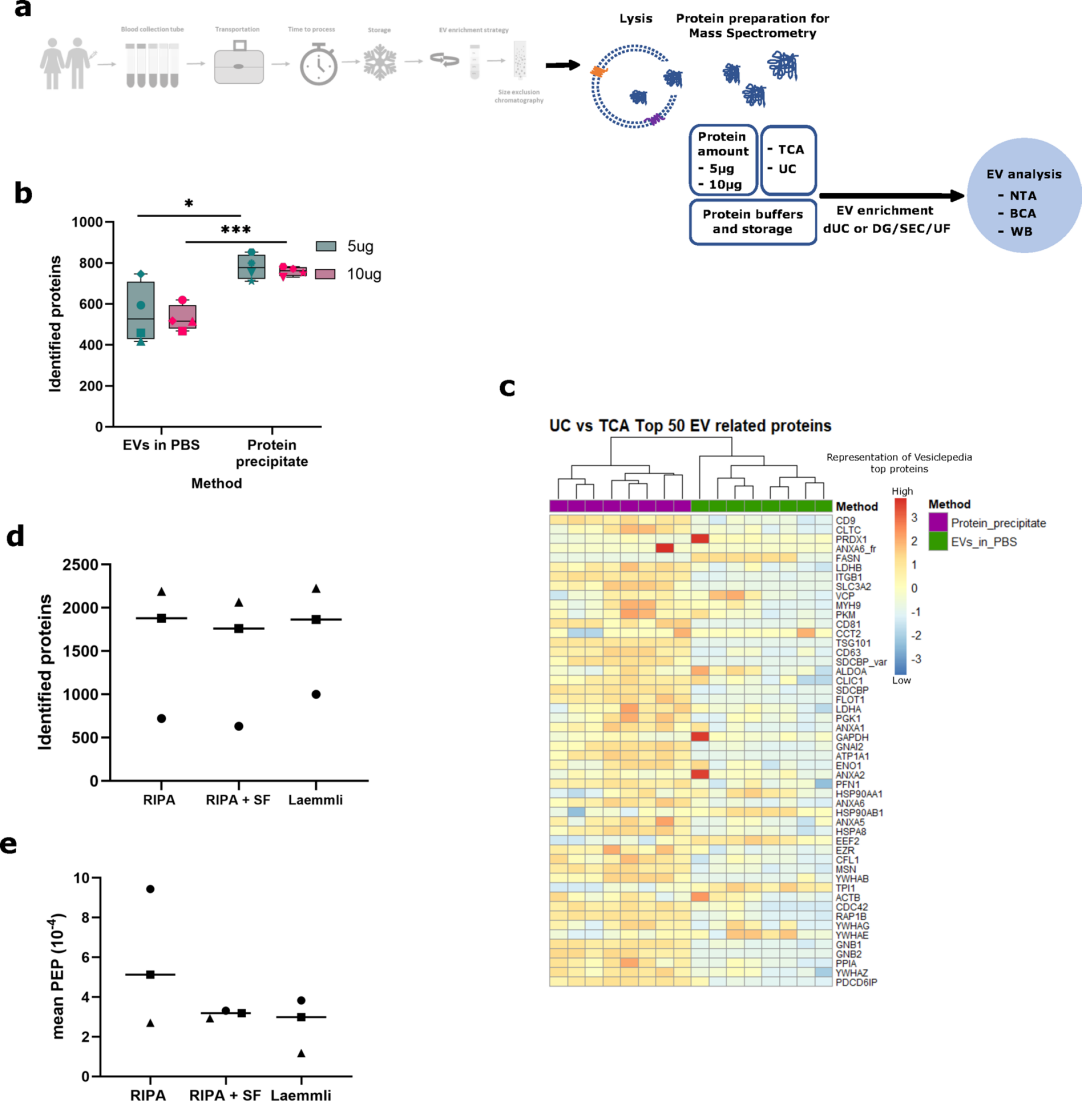
Optimization of EV lysis and protein preparation for mass spectrometry analysis. (**a**) Samples were collected from healthy volunteers or cancer patients (Figure 7d-e) in a Streck Cell-Free DNA BCT and processed either by differential or density gradient ultracentrifugation. Subsequently, SEC was performed and for DG samples, concentration was done by ultrafiltration. EV-enriched fractions were lysed and protein quantity was measured by bicinchoninic acid assay (BCA). (**b**) Protein lysates were pelleted, either by Trichloroacetic acid (TCA) precipitation or sedimentation by ultracentrifugation (UC), each in *n*=4 biological replicates, to assess protein identification by mass spectrometry. For both methods, 5 µg and 10 µg of protein input (acc. to BCA) were tested to identify the minimum amount of protein required for mass spectrometry analysis. Mean numbers of identified proteins are displayed together with 5^th^-to-95^th^ percentile range as box as well as a minimum to maximum range, and statistical significance is assessed using unpaired t test with *p=0.0310 (5 µg: precipitate vs. PBS) and ***p=0.0005 (10 µg: precipitate vs. PBS). (**c**) Heatmap of top 50 EV-related proteins (Vesiclepedia) to compare TCA precipitation and ultracentrifugation (UC), using MS data from *n*=4 biological replicates per condition, each of them split into two samples of 5–10 µg, respectively. One biological replicate for ultracentrifugation was collected in a Streck RNA BCT. EV enrichment in (**b**) and (**c**) was performed by differential ultracentrifugation and size exclusion chromatography. (**d**) Proteins identified by MS in 3 buffer conditions to resuspend protein pellets tested each in *n*=3 biological replicates (each symbol representing one sample). For RIPA + SF snap-freezing and storage of RIPA-lysed EVs was performed prior to MS submission. Mean values are displayed for each resuspension method. (**e**) Mean Posterior Error Probability (PEP) of proteins identified in (**d**). Mean values are displayed for each resuspension method. EV enrichment in (**d**) and (**e**) was performed by density gradient ultracentrifugation, followed by size exclusion chromatography and concentration by ultrafiltration. In (**b**), (**d**) and (**e**) symbols signify independent biological replicates.

Our optimization procedure aims at comprehensive proteomic analysis of patient plasma-derived EVs. Therefore, as a final step of pre-analytics, it is important to consider conditions of EV lysis and delivery to the mass spectrometry core facility (Fig. 7a).

EVs have been described to adhere to plastic surfaces like tubes and filters^30^. We therefore investigate the best way to recover EVs from the ultrafilter when performing lysis and protein precipitation immediately following EV purification. To this end, lysing within the filter yields higher amounts of EV-associated proteins than preceding transfer to a different tube (see Supplementary Fig. S5 online). Also, NTA confirms that particle numbers are reduced after ultrafiltration, compared with an aliquot collected right after the SEC, before concentrating (see Supplementary Fig. S5 online). The protein yield is further increased by using double protease inhibitor concentration (see Supplementary Fig. S7 online).

EVs can be delivered to the core facility in PBS with lysis immediately preceding mass spectrometry analysis. Alternatively, EVs can be lysed immediately following purification, resulting in delivery of the EV-derived protein to the mass spectrometry facility. We found that TCA protein precipitation immediately after lysis does not only lead to the identification of a somewhat higher number of proteins in mass spectrometry (Fig. 7b) but also a better representation of EV-related proteins as described in the Vesiclepedia database (Fig. 7c). EV purification from 9 to 10 mL of blood plasma regularly yields a total protein amount of 30–100 µg (see Supplementary Fig. S7 online). We therefore tested the protein amount required for mass spectrometry: analysis of 5 µg total protein input was equivalent to 10 µg in terms of proteins identified and EV representation of identified proteins (Fig. 7b-c).

Next, we tested different conditions of redissolution of the precipitated protein pellet (Fig. 7d): (1) RIPA: resuspension in RIPA buffer at the core facility, (2) RIPA + SF: resuspension in RIPA buffer immediately following TCA precipitation, snap-freezing, storage at −70 °C and transport to the core facility on dry ice, or (3) Laemmli: resuspension in Laemmli buffer at the core facility. While we see a tendency towards a higher number of identified proteins when precipitated pellets are redissolved at the core facility and do not go through the freeze-thaw process (Fig. 7d-e), according to the posterior error probability (PEP) there are no significant quality differences in the three conditions compared.

Therefore, we recommend lysis of EVs immediately after purification within the ultrafilter using double protease inhibitor concentration, followed by TCA precipitation.

## Discussion

In this study we optimize a protocol to study EV-derived proteomics in a clinical and translational setting.

We discover that among the BCTs tested, citrate shows a good profile with regard to high EV-derived protein yield and low hemolysis, while having the advantage of being readily available in the clinical practice. However, we detect an increase of CD41 signal in citrate tube-derived EVs, possibly corresponding either to an increase in platelet-derived EVs in citrate tubes^31^ or to activated platelet EVs^11^, both of which were reported previously. The impact of citrate on downstream analysis and on the isolation of CD41-positive EVs requires further evaluation. Among the other BCTs tested in our work, there are no significant differences. For consistency with the data already collected and to assess a single variable in each step of the proposed workflow, the usage of a single type of BCT was maintained throughout this work. Cell-free DNA BCT tubes were initially selected for this study according to preliminary data and considering that the cell stabilization properties in preservation BCTs make them good candidates for EV studies as reported in some studies for EV-derived nucleic acids^32^. As described above, results revealed that other BCTs might have less preanalytical impact for EV-derived proteomic studies. Thus, further assessment of the different steps of the workflow using other BCTs might offer additional insight.

In a clinical setting samples usually need to be transferred from the site of blood collection to the site of analysis, so we wanted to test the hypothesis that transportation of the samples at body temperature could help to stabilize blood cells and decrease ex vivo release of EVs which is a major concern for blood-derived EV studies. We found that both transportation conditions affect quantity and characteristics of EVs, but in a different manner. While transportation at 37 °C shows higher variance in particle numbers and a trend towards increased particle size, the transportation at ambient temperature produces an increase in non-physiological proteins. In line with previous reports, indicating that ambient temperature promotes platelet activation and degranulation^33,34^ our western blot analysis shows increased CD41. Analysis of mass spectrometric data shows enrichment in proteins related to platelet activation following transportation at room temperature. However, according to the PCA analysis the samples preserve their main characteristics irrespective of the transportation condition as they cluster by volunteer rather than by testing condition. On the other hand, the increased CD235a signal indicates that the main cell type affected by transportation at 37 °C are RBCs. EV size increase in the 37 °C condition could indicate an enrichment in RBC-derived large EVs (lEVs), the main carriers of Glycophorin a, and enrichment of neutrophil-derived proteins in this condition could be due to the interaction of RBC-derived lEVs with this cell type^35^. As the choice of anticoagulant impacts cell stability, the observed effects might also be anticoagulant-dependent. As a conclusion, whenever possible blood samples should be processed to platelet-poor plasma before transportation.

This is further confirmed by the increase in particle numbers, which we observe with increasing needle-to-processing time. Those non-physiological particles contain altered protein profiles with a shift towards cell-specific markers that might be related to EV release during cell activation or apoptosis. Our results are consistent with a previously published mass spectrometry analysis that compared blood processing intervals with different anticoagulants^11^.

Following immediate processing of full blood, we find that storage temperature of platelet-poor plasma does not significantly impact on quantity and quality of isolated EVs. However, this result might have to be further refined in the future with an increased number of biological replicates.

In general, the limited number of male study participants does not allow to assess for sex differences. In the future, it might be necessary to investigate the observed effects with particular regard to intersexual differences.

Another point of potential refinement for future experiments is the number of replicates of Western Blot and mass spectrometry analyses in order to further shed light on the composition of the plasma EV-derived proteome in response to the various protocol changes.

The aim of this study is the development of an EV isolation protocol that can be implemented in the clinical and translational setting and allows upscaling to a high sample throughput for comprehensive biomarker identification. Here, the combination of DGUC with SEC outperforms differential ultracentrifugation followed by SEC. The EV population isolated with DGUC shows higher yield and purity than dUC-processed EVs. High-density lipoproteins can mimic extracellular vesicles in NTA and accordingly, dUC-isolated EVs show increased apolipoprotein signal while DGUC produces higher signal of EV-related markers. Consequently, the combination of DGUC and SEC yields highly enriched EVs, laying the grounds for identification of EV-derived biomarkers. Another advantage of DGUC is the purification of a comprehensive assembly of EV populations, while dUC separates small and large EVs which then need to be re-combined or further processed separately, thereby significantly increasing work load in the laboratory and hampering upscaling of the method.

Size exclusion chromatography is a common second step for separation of EVs from blood components of similar density. In this study we show that home-made glass columns using Sepharose CL-4B are a reliable and sustainable alternative to commercial SEC columns. Even more, the use of home-made columns increases cost-effectiveness over time and circumvents delivery shortages and batch effects of commercial SEC columns. Finally, we demonstrate that following SEC, ultrafiltration is a more efficient method than another round of ultracentrifugation for collection and concentration of EVs. Due to the potential trap of vesicles in the filter, it is advisable to perform the lysis directly in the ultrafiltration unit, rather than transferring the volume to a new tube for lysis.

When working with extracellular vesicles, especially with patient samples, the material is precious and the protein yield limiting. Interestingly, the submission of 5 µg of EV-derived protein, corresponding to the protein yield from about 1 mL of full blood, is sufficient for comprehensive mass spectrometry analysis. Regarding submission to the core facility, protein precipitation is advantageous to keeping EVs dissolved in PBS, possibly due to effects of tube wall adherence and succeeding loss of unlysed vesicles^36^. Mass spectrometry analyses are usually not performed within the clinical facility, and there is no clear recommendation on how to preserve the EV-enriched samples during time to analysis. Our results indicate a tendency towards higher number of proteins identified by mass spectrometry when storing the precipitated protein at 4 ° rather than snap-freezing and thawing the EV lysate, which might be due to protein degradation. Accordingly, for reasons of practicability, sustainability and protein recovery, we recommend TCA precipitation of EV-derived protein, storage of pellets at 4 °C and redissolution in RIPA or Laemmli buffer at the core facility preceding MS analysis.

While this protocol aims at providing sufficient experimental details for comprehensive plasma-derived EV proteome analysis, it is true that also in our lab, inconsistencies in handling had to be overcome and practical experience with EV handling and purification is needed in order to produce reliable and high quality data.

For future experiments, the number of replicates of Western Blot and mass spectrometry analyses could be increased in order to further shed light on the composition of the plasma EV-derived proteome in response to the various protocol changes.

In this study we evaluate pre-analytical variables that could act as confounding factors in proteomic blood-derived EV studies, and suggest an optimized protocol considering blood collection, sample transportation, time to process, storage, enrichment methods and protein preparation for mass spectrometry analysis.

## Materials and methods

### Ethics approval and consent to participate

Ethics Approval was obtained from the Ethics Comitee of the University of Heidelberg, Medical Faculty Mannheim (Ethics Approval No. 2014-598N-MA). Informed consent was obtained from all participants. All methods were performed in accordance with the Declaration of Helsinki.

### Plasma isolation

Peripheral blood was collected from two male and seven female healthy individuals aged 20 to 43 years by standard venipuncture using a Vacuette Safety blood collection set with a 21G needle (Greiner Bio-one, Frickenhausen, Germany). Each blood draw followed by independent processing was considered a biological replicate as outlined in the figure legends. For experiments in Fig. 7d-e isolated EVs from cancer patients aged 35–83 years were used. Informed consent was obtained from all participants. Blood was drawn directly into 10.0 mL Cell-free DNA BCT (Streck, La Vista, NE, USA). For the comparison of blood collection tubes (Fig. 2), we additionally used 9.0 mL EDTA K3E S-Monovette (Sarstedt, Nümbrecht, Germany), 10.0 mL Citrate 9NC 0.106 mol/l S-Monovette (Sarsted, Nümbrecht, Germany), 9 mL ACD-A Vacuette (Greiner Bio-one, Frickenhausen, Germany) and 10.0 mL RNA Complete BCT (Streck, La Vista, NE, USA). All tubes were filled completely until the vacuum was exhausted. The tubes were inverted immediately after blood collection according to manufacturer´s recommendations. To produce platelet-poor plasma (PPP), tubes were centrifuged at 2,500 *xg* for 15 min at room temperature (15–20 °C). Then the plasma was collected from the top with a Pasteur pipette (down to around 0.5–1.0 mL above the buffy coat) and placed in a new 15 mL tube. The collected plasma was centrifuged a second time at 2,500 *xg* for 15 min at room temperature, and again the plasma was collected from the top down to 0.5 mL above cell pellet and either used immediately for an experiment or placed in a 5 mL cryotube until further processing. Storage temperature was − 20 °C unless otherwise specified (see Supplementary Fig. 4 online). All further procedures were carried out in low-bind tubes (Sarsted, Nümbrecht, Germany), in order to avoid EV adhesion to the tube wall.

### Sample transportation

For experiments assessing the impact of temperature during sample transportation (Fig. 3), peripheral blood samples were collected at University Hospital Mannheim and transferred to the research laboratory within the next two hours using a motorized vehicle (about 15 min drive). The tubes transported at 37 °C were entered into a Mini Bio Isotherm Hot container (M.&G. Intl srl, Italy) immediately after blood draw and then transported as described above. For the immediate processing condition, platelet-poor plasma was produced by tandem centrifugation as detailed above immediately after blood draw and before transportation. All tubes from the same experiment were transported together to the research laboratory.

### Hemolysis

Hemolysis was quantified from a 2µL PPP aliquot using a NanoDrop One Microvolume UV-Vis Spectrophotometer (Thermo Fischer Scientific, Waltham, MA, USA) by measuring sample absorbance at 414 nm. A standard curve was generated starting from a fully lysed red blood cell sample, and serial dilutions were made from 100 to 0.025%. Each sample hemolysis value was calculated according to the linear range of the standard curve.

### Differential ultracentrifugation (dUC)

dUC, followed by SEC, for isolation of EVs from PPP was used for the analysis of optimal needle-to-processing time (Fig. 4) and for comparison to density gradient ultracentrifugation (Fig. 5). PPP was poured into an ultracentrifuge tube (Seton, S5031 or Beckmann Coulter C140303) before addition of 0.22 µM-filtered PBS to reach a total volume of 10 mL. Tubes were centrifuged at 10,000 *xg* for 20 min at 4 °C in a Beckmann Optima XE-90 ultracentrifuge (Beckmann Coulter GmbH, Krefeld, Germany) using a SW 40 Ti swinging bucket rotor. Supernatant was transferred to a new ultracentrifugation tube to be centrifuged at 100,000 *xg* for 2 h at 4 °C in the ultracentrifuge as specified above. Resulting 100,000 *xg* pellets were resuspended in 100 µL of 0.22 µM-filtered PBS, stored at 4 °C overnight and entered into a SEC column the next day.

### Density gradient ultracentrifugation (DGUC)

EVs were purified from PPP by DGUC, size exclusion chromatography (SEC) and ultrafiltration (UF) unless otherwise specified. For DGUC, a discontinuous density gradient was prepared using a 60% iodixanol solution (Optiprep, Abcam, United States) according to *Dhondt et al. 2020*^37^. Briefly, 2 mL of iodixanol solution 40% (w/v) were layered on the bottom of an ultracentrifugation tube (Seton S5031 or Beckmann Coulter C140303), followed by 2 mL of 20% (w/v), 2 mL of 10% (w/v) and 1.5–2 mL of 5% (w/v). 3–4 mL of PPP were layered on the top of the gradient. When stored, PPP was thawed in a water bath at 37 °C before entering the gradient. DGUC was performed at 100,000 *xg* for 16 h at 4 °C in a Beckmann Optima XE-90 ultracentrifuge (Beckmann Coulter GmbH, Krefeld, Germany) using a SW 40 Ti swinging bucket rotor. A visible band between 40% and 20% gradients (high-density band^38^) was collected and the resulting EV-enriched volume of about 1 mL was transferred to a 1.5 mL tube.

### Size exclusion chromatography (SEC) and ultrafiltration (UF)

For SEC following dUC, we used single qEV 35 nm legacy columns (Izon, SO6, Christchurch, New Zealand) according to the manufacturer’s instructions. Briefly, columns were allowed to reach room temperature for at least 30 min and then washed with at least one column volume of 0.22 μm-filtered PBS. 100–150 µL of resuspended pellet volume resulting from the 100,000 *xg* ultracentrifugation step were allowed to enter the column before addition of 600 µL of 0.22 μm-filtered PBS and collection of the void volume (800 µL). Subsequently, seven 200 µL fractions were collected separately. A 10 µL aliquot from each fraction was used for NTA (nanoparticle tracking analysis) measurement. All fractions with valid particle measurements in NTA were lysed (see EV lysis) and pooled for subsequent protein precipitation.

For SEC following DGUC we used qEV2 35 nm legacy columns (Izon, SP8, Christchurch, New Zealand) according to manufacturer´s instructions. Briefly, columns were allowed to reach room temperature for at least 30 min and then washed with at least one column volume of 0.22 μm-filtered PBS. The EV-enriched sample resulting from DGUC was loaded onto the column, its tube was washed with 1 mL of 0.22 μm-filtered PBS and this volume was also loaded onto the column immediately. Additional 14 mL of 0.22 μm-filtered PBS were added to the column and subsequently collected as void volume. Then, 5 mL of filtered PBS were entered into the column and collected in an Amicon Ultra-15 Centrifugal filter 10 kDa (Merck Millipore, Darmstadt, Germany) (EV-enriched fraction). The Amicon tube was centrifuged at 3,683 *xg* at 4 °C for 20 min. 10 µL of this concentrated volume were aliquoted for NTA and 40 µL for transmission electron microcopy (TEM). The remaining volume of the EV-enriched fraction was lysed immediately by addition of lysis buffer to the Amicon filter.

For comparison of different types of SEC columns (Fig. 6), self-made Sepharose CL-4B and Sephacryl S300 columns were prepared as follows:

Before use, Sepharose CL-4B (CL4B200, Sigma-Aldrich, USA) and Sephacryl S300 (S300HR, Sigma-Aldrich, USA) were swollen with 0.22 μm-filtered PBS rolling for one hour at 4 °C. Afterwards, bottles were left standing for at least three hours to allow gravity sedimentation of the resins. When completely settled, part of the buffer was removed to have a 70% (v/v) resin slurry. The slurry was resuspended carefully until homogeneous and 28 mL of slurry were layered carefully into a glass chromatography Econo-Column 1.5 × 20 cm (Bio-Rad Laboratories, Feldkirchen, Germany), aiming at 20 mL of stacked resin. The columns were left to settle overnight at 4 °C and additional volume was added the next day if necessary. Before entering a sample, the columns were washed with two column volumes of 0.22 μm-filtered PBS.

Elution profiles of Sepharose CL-4B and Sephacryl S300 columns (Fig. 6e, see Supplementary Fig. S6 online) were assessed using 400 µL of dextran blue 2 g/L (gel filtration calibration kit HMW, Cytiva, USA), and in a separate run, using a protein mix of 350 µL thyroglobulin 2 g/L (Gel filtration calibration kit HMW, Cytiva, USA) and 50µL of bovine serum albumin 4 g/L (Carl Roth, Germany). For qEV2 35 nm legacy columns (Izon, New Zealand) 900 µL dextran blue and a protein mix of 800 µL thyroglobulin 2 g/L and 100 µL bovine serum albumin 4 g/L were used.

After entering the corresponding standard, 1 mL fractions were collected until completing at least one column volume. Dextran blue elution profile was assessed by measuring the absorbance of each fraction at 595 nm in a microplate using a Tecan Infinite F200 (Tecan Trading AG, Switzerland). For the protein mix elution profile, 150 µL of each fraction were mixed with 150 µL of Bradford reagent 5x (SERVA, Heidelberg, Germany) diluted to 1x with ddH_2_O in a microplate, and absorbance was measured at 595 nm using a Tecan Infinite F200 (Tecan Trading AG, Switzerland). After collecting the sample fractions, columns were washed with two column volumes of 0.22 μm-filtered PBS, 1 mL of NaOH 0.5 M, and two column volumes of PBS containing 0.05% NaN_3_, and stored at 4 °C until further use.

For peripheral blood samples (Figs. 6b-d, f-g, see Supplementary Fig. S6 online), plasma preparation and DGUC were performed as described above. EV-enriched samples collected from DGUC were entered into a Sepharose CL-4B/Sephacryl S300 column (prepared as above). Then 6 mL of 0.22 μm-filtered PBS were loaded and 6 mL collected for void volume, followed by 5 mL for EV-enriched fractions, followed by 4 mL for non-EV-enriched control fractions. For Fig. 6b-d EV-enriched eluates were subjected to bulk analysis and further processed as described above for the qEV2 35 nm legacy columns. For analysis of elution profiles, EV-enriched or non-enriched fractions were collected per mL and analyzed using Western Blot (Fig. 6f-g). The SEC fractions with EV marker protein signals were then pooled before TCA precipitation and subsequent Western Blot (see Supplementary Fig. S6 online).

### Nanoparticle tracking analysis (NTA)

Particle concentration and size distribution from EV-enriched fractions were measured using a ZetaView PMX-230 Twin (Particle Metrix GmbH, Inning am Amersee, Germany) and ZetaView version 8.05.16 SP7 software. A sample aliquot was diluted 1:100 to 1:100,000 in DPBS (Sigma Aldrich, Darmstadt, Germany). The following instrument settings were used: laser wavelength 488 nm, filter wavelength scatter, sensitivity 80 and shutter 100. For each sample, 21 s video was recorded with at least 8 frames used for analysis.

### EV Lysis and protein precipitation

Isolated EVs were lysed using RIPA buffer (Tris-Hcl pH 7.6 25 mM, NaCl 150 mM, sodium deoxycholate 0.1%, SDS 0.1%, Triton X-100 0.1%, sodium orthovanadate 2 mM, NaF 50 mM, protease inhibitor tablets (Pierce protease inhibitor Mini tablets, Thermo Scientific, USA) 5%, phosphatase inhibitor tablets (PhosSTOP EASYpack, Roche, Germany) 2%). One volume of 5x buffer was added to four volumes of sample, vortexed for 30 s and incubated on ice for 30 min. Lysate was cleared at 20,817 *xg* (Centrifuge 5430R, Eppendorf, Germany) for 10 min at 4 °C and pellets were discarded. 35 µL of cleared lysate were aliquoted for BCA protein measurement. Lysate volume was split according to each experiment and 1 volume of trichloroacetic acid (TCA) was added to 9 volumes of lysate and left to precipitate overnight at 4 °C. The next day, tubes with protein precipitate were centrifuged at 20,817 *xg* and 4 °C for 5 min and supernatant was removed and discarded without disturbing the protein. The pellet was washed with 1000 µL of cold acetone, centrifuged again at 20,817 *xg* and 4 °C for 5 min and supernatant was discarded. In total, three acetone washes were performed. After the third acetone wash, the pellet was dried in a Vacuum Concentrator (5301, Eppendorf, Germany) at 30 °C for 20–25 min. After visual verification of a completely dry protein pellet without any acetone traces, pellets were sealed and stored at 4 °C until western blot or mass spectrometry analysis.

In Supplementary Fig. S7 online, 400–550 µL of purified EVs in 0.22 μm filtered PBS were split evenly into the different conditions. Inhibitor concentrations were modified as specified in the figure legend with 2x phosphatase inhibitor corresponding to a phosphatase inhibitor concentration of 4%, 2x protease inhibitor corresponding to a protease inhibitor concentration of 10%, and 50% volume increase corresponding to the supplementation of the sample with half its volume of buffer (0.22 μm-filtered PBS).

In Fig. 7b-c, EV lysate volumes corresponding to 5–10 µg of protein, respectively, were either TCA-precipitated in a 1.5 mL tube as described above or diluted in 8 mL of 0.22 μm-filtered PBS and pelleted in a Beckmann Optima XE-90 ultracentrifuge (Beckmann Coulter GmbH, Krefeld, Germany) at 100,000 *xg* for 2 h at 4 °C. Supernatant was decanted by gentle inversion of the tube and the remaining drops in the tube opening where removed. The pellet was resuspended in the volume remaining in the tube, and sealed. Tubes were stored at 4 °C no more than 5 days and then transferred to the mass spectrometry core facility. Corresponding UC tubes and TCA precipitates were transported on ice.

For Fig. 7d-e, EV-derived protein lysates were divided into three equal volumes and then TCA-precipitated. One of the protein pellets was resuspended immediately with RIPA 1x buffer (described above) and snap-frozen in liquid nitrogen for storage at −70 °C. Later, this sample was transferred to the mass spectrometry core facility on dry ice. The other two protein precipitates were stored at 4 °C and transferred to the mass spectrometry core facility on ice. Upon arrival at the core facility, they were resuspended in Laemmli 1x (see Western Blotting, below) or in RIPA buffer (Thermo Scientific, 89900). In all three conditions, protein pellets for this experiment were resuspended to a final concentration of 0.19–0.25 µg/µL. The concentration was identical in the different conditions of one replicate.

### Bicinchoninic acid (BCA) for protein quantification

Micro BCA Protein assay (Thermo Fischer Scientific, Rockford, Illinois, USA) was performed for protein quantification according to the manufacturer’s instructions. Briefly, a bovine serum albumin (BSA) standard curve of 0.5 µg/mL – 200 µg/mL was prepared from a 2 mg/mL stock. The assay was performed in a microtiter plate. For each sample well 135 µL of 0.22 μm-filtered PBS and 15 µL of sample were mixed thoroughly. For the standard curve, 150 µL of the respective standard solution were used. Subsequently, 150 µL of BCA working reagent were added per well and mixed thoroughly. The plate was sealed with an adhesive foil and incubated for 2 h at 37 °C. Hence, absorbance at 562 nm was measured using a Tecan Infinite F200 (Tecan Trading AG, Switzerland), and the protein concentration of each sample was calculated according to the standard curve.

### Western blotting

1x Laemmli (Tris-HCl pH 6.8 62.5 mM, SDS 2%, Glycerol 5%, Beta-Mercaptoethanol 2%, Bromophenol blue 0.1%) was used to redissolve protein pellets for immunoblotting. Redissolved protein samples were either loaded to an SDS gel immediately or stored at −20 °C until further use.

Samples redissolved in Laemmli were boiled at 95 °C for 4 min. Equal sample volumes were run on home-made SDS-PAGE gels and transferred to a PVDF membrane with 0.45 μm pore size (Immobilon-P, Merck Millipore, Germany). After Ponceau staining, membranes were rinsed with PBST (PBS with 1% Tween-20), incubated first in 5% skim milk PBST for 60 min and then in primary antibody solution in 5% skim milk PBST overnight at 4 °C while rotating. Membranes were washed three times, 15 min each, in PBST, incubated in secondary antibody solution (1:10,000 in 5% skim milk PBST) for one hour at room temperature, then washed again three times for 5–15 min. For Hsc70 detection TBST (Tris 20 mM, NaCl 150 mM, pH 7.6, Tween-20 1%) was used instead of PBST. Chemiluminescence was detected using SuperSignal West pico & femto ECL reagents (Thermo Fischer Scientific, Life Technologies, USA) and a ChemiDoc imaging System (Bio-Rad Laboratories, Feldkirchen, Germany) with an exposure time of 1–300 s. Image analysis and quantification were performed using Image Lab Software version 6.1.0 (Bio-Rad Laboratories, Feldkirchen, Germany).

### Antibodies

The following primary antibodies were used for immunoblotting: CD9 (Cell Signalling Technology, #13403, 1:1,000), GAPDH (Proteintech, #60004-1, 1:1,000), ALIX (abcam, ab275377, 1:1,000), ApoA1 (R&D Systems, MAB36641, 1:2,500), ApoB (R&D Systems, MAB41242, 1:1,000), CD235a (Invitrogen, JC159, 1:500), CD41 (Integrin α−IIb, abcam, ab83961, 1:1,000), Hsc70 (abcam, ab51052, 1:2,500). The following secondary antibodies were used: Peroxidase conjugated AffiniPure goat anti-mouse IgG (Jackson ImmunoResearch Laboratories, 115-035-003, 1:10,000) and Peroxidase conjugated AffiniPure goat anti-rabbit IgG (Jackson ImmunoResearch Laboratories, 111-035-003, 1:10,000).

### Transmission electron microscopy

Concentrated EV isolates were adsorbed onto glow discharged carbon coated grids, washed in aqua bidest and negatively stained with 2% aqueous uranyl acetate. Micrographs were taken with a Zeiss EM 910 or EM 912 at 100 kV (Carl Zeiss, Oberkochen, Germany) using a slow scan CCD camera (TRS, Moorenweis, Germany).

### Mass spectrometry

For experiments in Fig. 7b-c a protein amount of 5–10 µg was digested using the SP3 protocol and resulting peptides were dried and stored frozen pending the LC-MS/MS measurement^39^. For experiments in Figs. 3 and 7d-e a protein amount of 10 µg was digested (trypsin) using an AssayMAP Bravo liquid handling system (Agilent technologies) running the autoSP3 protocol according to Müller et al.^40^. Resulting peptides were dried and stored frozen pending the LC-MS/MS measurement.

For experiments in Fig. 7b-c LC-MS/MS analysis was carried out on an Ultimate 3000 UPLC system (Thermo Fisher Scientific) directly connected to an Orbitrap Exploris 480 mass spectrometer for a total of 60 min. Peptides were online desalted on a trapping cartridge (Acclaim PepMap300 C18, 5 μm, 300Å wide pore; Thermo Fisher Scientific) for 3 min using 30 µl/min flow of 0.05% TFA in water. The analytical multistep gradient (300 nl/min) was performed using a nanoEase MZ Peptide analytical column (300Å, 1.7 μm, 75 μm x 200 mm, Waters) using solvent A (0.1% formic acid in water) and solvent B (0.1% formic acid in acetonitrile). For 42 min the concentration of B was linearly ramped from 4 to 30%, followed by a quick ramp to 78%, after two minutes the concentration of B was lowered to 2% and a 10 min equilibration step appended. Eluting peptides were analyzed in the mass spectrometer using data independent acquisition (DIA) mode. A MS1 scan at 120 k resolution (380–1400 m/z, 300% AGC target, 45 ms maxIT) was followed by 20 DIA scans of variable width covering a mass range of 400–1000 m/z with 0.5 Da overlap between the windows. Scans were recorded at 30 k resolution (1,000% AGC target, 54 ms maxIT) and a fragmentation energy of 28%. Each sample was followed by a wash run (40 min) to minimize carry-over between samples. Instrument performance throughout the course of the measurement was monitored by regular (approx. one per 48 h) injections of a standard sample and an in-house shiny application.

For experiments in Figs. 3 and 7c-d the LC-MS/MS analysis was carried out on an Vanquish Neo UPLC system (Thermo Fisher Scientific) directly connected to an Orbitrap Exploris 480 mass spectrometer for a total of 60 min. Peptides were online desalted on a trapping cartridge (Acclaim PepMap300 C18, 5 μm, 300Å wide pore; Thermo Fisher Scientific) at a flow rate of 30 µl/min of 0.05% TFA in water for a total loading volume of 60 µl. The analytical multistep gradient (300 nl/min) was performed using a nanoEase MZ Peptide analytical column (300Å, 1.7 μm, 75 μm x 200 mm, Waters) using solvent A (0.1% formic acid in water) and solvent B (0.1% formic acid in acetonitrile). For 42 min the concentration of B was linearly ramped from 4 to 30%, followed by a quick ramp to 78%, after two minutes the concentration of B was lowered to 2% and an equilibration step of 3x the column volume was appended. Eluting peptides were analyzed in the mass spectrometer using data independent acquisition (DIA) mode. A MS1 scan at 120 k resolution (380–1400 m/z, 300% AGC target, 45 ms maxIT) was followed by 20 DIA scans of variable width covering a mass range of 400–1000 m/z with 0.5 Da overlap between the windows. Scans were recorded at 30 k resolution (1,000% AGC target, 54 ms maxIT) and a fragmentation energy of 28%. Each sample was followed by a wash run (40 min) to minimize carry-over between samples. Instrument performance throughout the course of the measurement was monitored by regular (approx. one per 48 h) injections of a standard sample and an in-house shiny application.

### Data analysis

Analysis workflows for mass spectrometry data were carried out in two slightly differing protocols, that are detailed separately below.

For experiments displayed in Fig. 7b-c analysis of DIA RAW files was performed with Spectronaut (Biognosys, version 17.1.221229.55965) in directDIA+ (deep) library-free mode. Default settings were applied with the following adaptions. Within the *Pulsar Search* in *Peptides* the *Max Peptide Length* was set to 35, in *Result Filters* the *Peptide Charge* was enabled and the *Max Charge* set to 6 and the *Min Charge* set to 2. Within *DIA Analysis* under *Identification* the *Precursor PEP Cutoff* was set to 0.01, the *Protein Qvalue Cutoff (Run)* set to 0.01 and the *Protein PEP Cutoff* set to 0.05. In *Quantification* the *Proteotypicity Filter* was set to *Only Protein Group Specific*, the *Corss-Run Normalization* was disabled, the *Quantification window* was set to *Not Synchronized* and the *Major Group Quantity* was set to *Sum peptide quantity*. The data was searched against the human proteome from Uniprot (human reference database with one protein sequence per gene, containing 20,591 unique entries from first of March 2023) and the contaminants FASTA from MaxQuant (246 unique entries from twenty-second of December 2022).

For experiments displayed in Figs. 3 and 7d-e sample handling (sample preparation and LC-MS/MS analysis) have been performed in a block randomization^41^ order.

Analysis of DIA RAW files was performed with Spectronaut (Biognosys, version 18.6.231227.55695) in directDIA+ (deep) library-free mode. Default settings were applied with the following adaptions. Within *DIA Analysis* under *Identification* the *Precursor PEP Cutoff* was set to 0.01, the *Protein Qvalue Cutoff (Run)* set to 0.01 and the *Protein PEP Cutoff* set to 0.01. In *Quantification* the *Proteotypicity Filter* was set to *Only Protein Group Specific*, the *Protein LFQ Method* was set to *MaxLFQ* and the *Quantification window* was set to *Not Synchronized (SN 17)*. The data was searched against the human proteome from Uniprot (human reference database with one protein sequence per gene, containing 20,597 unique entries from ninth of February 2024) and the contaminants FASTA from MaxQuant (246 unique entries from twenty-second of December 2022).

Analyses of mass spectrometry data for the transportation experiment (Fig. 3) were performed using R version 4.1.3 and Metascape^42^ (http://metascape.org). Heatmap for Fig. 7c was elaborated using the pheatmap function in R.

In general, data management and calculations were performed using GraphPad Prism 10 (Graphpad, Software Inc., La Jolla, CA, USA). Comparisons between two groups were performed using the Wilcoxon matched-pairs signed-rank test and paired or unpaired t-test (as indicated). For comparison using fold changes, the binary logarithms were calculated and one sample t-tests performed. Statistical significance for the performance of blood preservation tubes was always tested against EDTA values and subsequently the p-value was adjusted for multiplicity using the Bonferroni-Holm method. p-values of < 0.05 were considered statistically significant, with p-values represented as follows: * *p* < 0.05, ** *p* < 0.01, *** *p* < 0.001, and **** *p* < 0.0001. “ns” denotes differences in means that were not significant. All error bars shown represent standard deviation if not stated otherwise.

## Electronic supplementary material

Below is the link to the electronic supplementary material.

Supplementary Material 1

Supplementary Material 2

Supplementary Material 3

Supplementary Material 4

## Data Availability

Mass spectrometry data that supports the findings of this study has been uploaded to the PRIDE server and is available under the following accession code: PXD058373 (https://www.ebi.ac.uk/pride/archive/projects/PXD058373).
