# Supplementary material for: A translational protocol optimizes the isolation of plasma-derived extracellular vesicle proteomics
Source: Sci Rep. 2025 Jul 7;15:24292. doi: 10.1038/s41598-025-08366-8 (PMC12234866; doi:10.1038/s41598-025-08366-8)
Supplement: Supplementary file 4 — Supplementary Material 4 [file 41598_2025_8366_MOESM4_ESM.pdf]

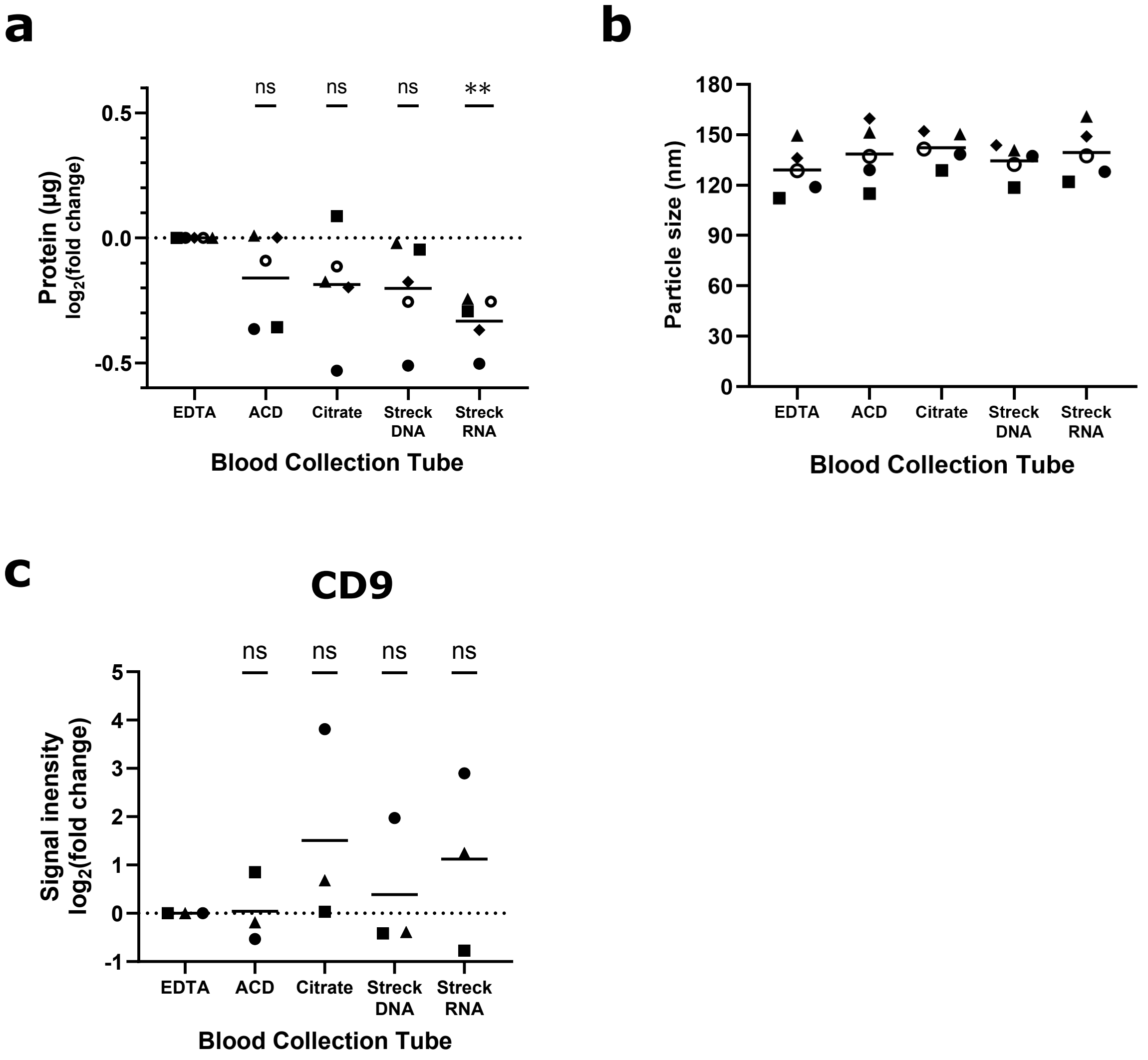

Supplementary Figure S1

Supplementary Figure S1: Differential effect of blood collection tubes (BCTs)

a. Total protein obtained in EV-enriched fractions from each BCT after lysing EV enriched fractions, measured by BCA. Data normalized to the reference BCT (EDTA). Mean of normalized value is displayed for each BCT and statistical significance is assessed using one sample t test (Bonferroni-Holm-adjusted p-values) with p=0.2562 (ACD), p=0.2562 (Citrate), p=0.2529 (Streck DNA) and **p=0.0088 (Streck RNA)

b. Median size of the particles recovered in EV-enriched fractions from the different BCTs, measured by NTA.

c. Immunoblotting quantification of CD9. Data normalized to the reference BCT (EDTA). Mean of normalized value is displayed for each BCT and statistical significance is assessed using one sample t test with p=0.9264 (ACD), p=0.3253 (Citrate), p=0.6718 (Streck DNA) and p=0.4005 (Streck RNA).

Data in a-b is derived from n=5 biological replicates, and c from n=3 biological replicates.

Symbols signify independent biological replicates corresponding with Figure 2.

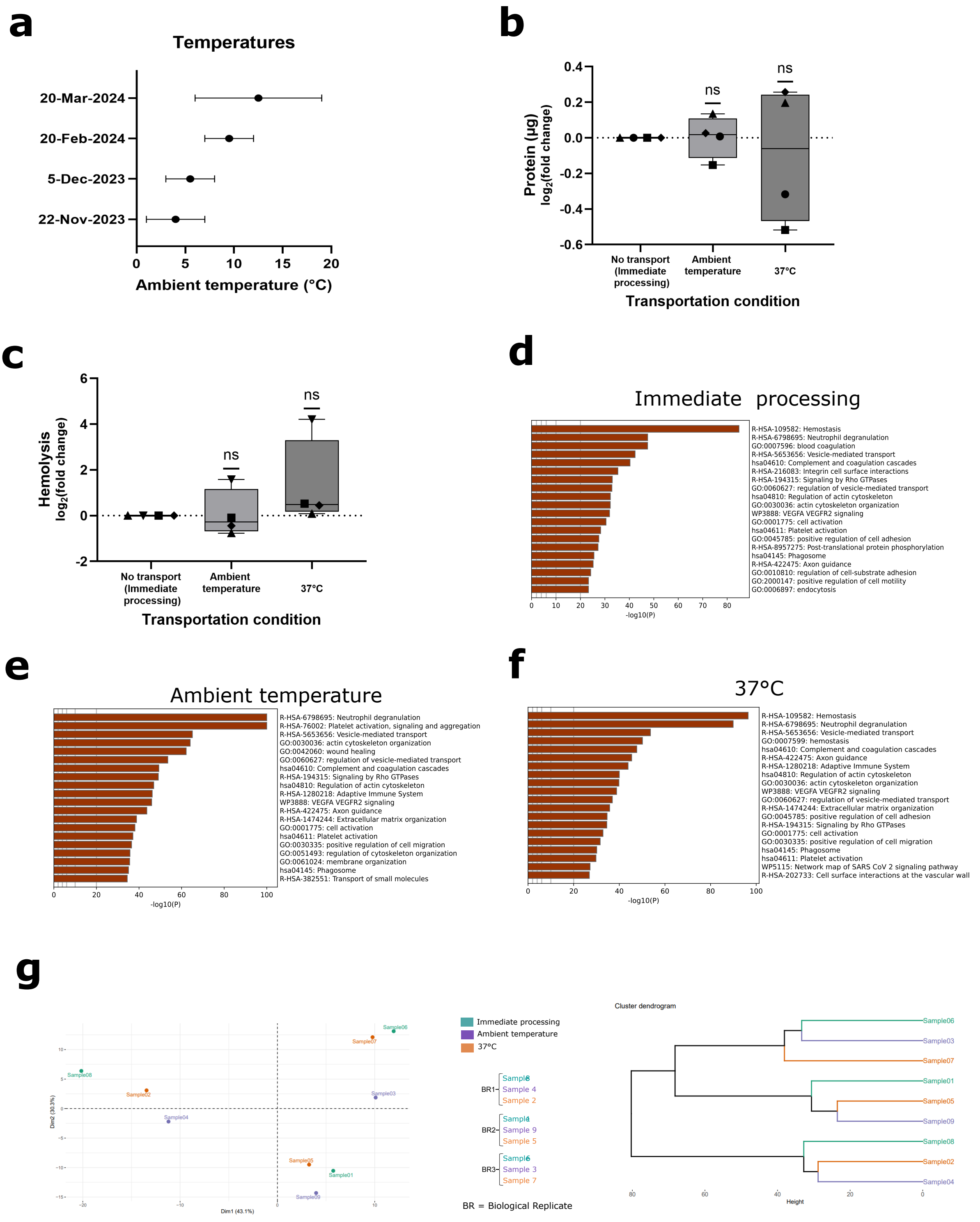

Supplementary Figure S2

Supplementary Figure S2: Influence of sample transportation temperature

a. Record of the ambient temperatures on the dates of each biological replicate of the transportation experiment.

b. Total protein obtained from each transportation condition after lysing EV-enriched fractions, measured by BCA. Data normalized to the no transport (immediate processing) condition. Median of normalized value is displayed for each transportation condition and statistical significance is assessed using one sample t test with p=0.9504 (Ambient temperature) and p=0.6512 (37°C).

c. Hemolysis of the platelet-poor plasma obtained from the different conditions, measured by absorbance at 414nm. Data normalized to the no transport (immediate processing) condition. Median of normalized value is displayed for each transportation condition and statistical significance is assessed using one sample t test with p=0.900 (Ambient temperature) and p=0.2673 (37°C).

d-f. Top 20 functional categories identified in the mass spectrometry protein sets of the three transportation conditions according to Metascape enrichment analysis.

g. Mass spectrometry analysis of EV-derived proteins after transportation at different temperatures: principal component analysis (PCA) and hierarchical clustering.

Data in b-c is derived from n=4 biological replicates. Data in d-g is derived from n=3 biological replicates.

Symbols signify independent biological replicates corresponding with Figure 3.

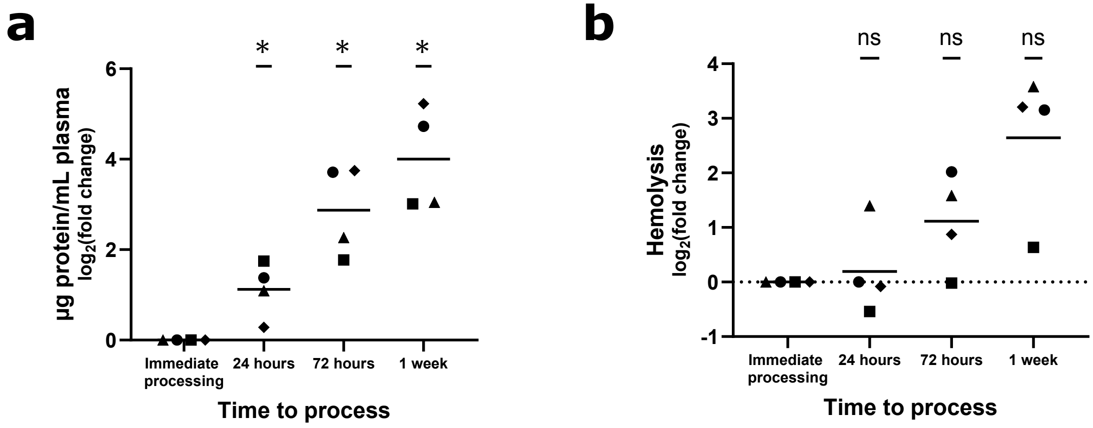

Supplementary Figure S3

Supplementary Figure S3: Relevance of needle-to-processing time

a. EV-derived protein amount per mL plasma at different processing timepoints, measured by BCA. Data normalized to the immediate processing condition. Mean of normalized value is displayed for each time condition and statistical significance is assessed using one sample t test (Bonferroni-Holm-adjusted p-values) with *p=0.0366 (24 hours), *p= 0.0214 (72 hours), *p=0.0180 (1 week).

b. Hemolysis measurement of platelet-poor plasma at different processing timepoints, measured by absorbance at 414nm. Data normalized to the immediate processing condition. Mean of normalized value is displayed for each time condition and statistical significance is assessed using one sample t test (Bonferroni-Holm adjusted p-values) with p=0.6747 (24 hours), p=0.1756 (72 hours) and p=0.0891 (1 week).

Data in a and b is derived from n=4 biological replicates.

Symbols signify independent biological replicates corresponding with Figure 4.

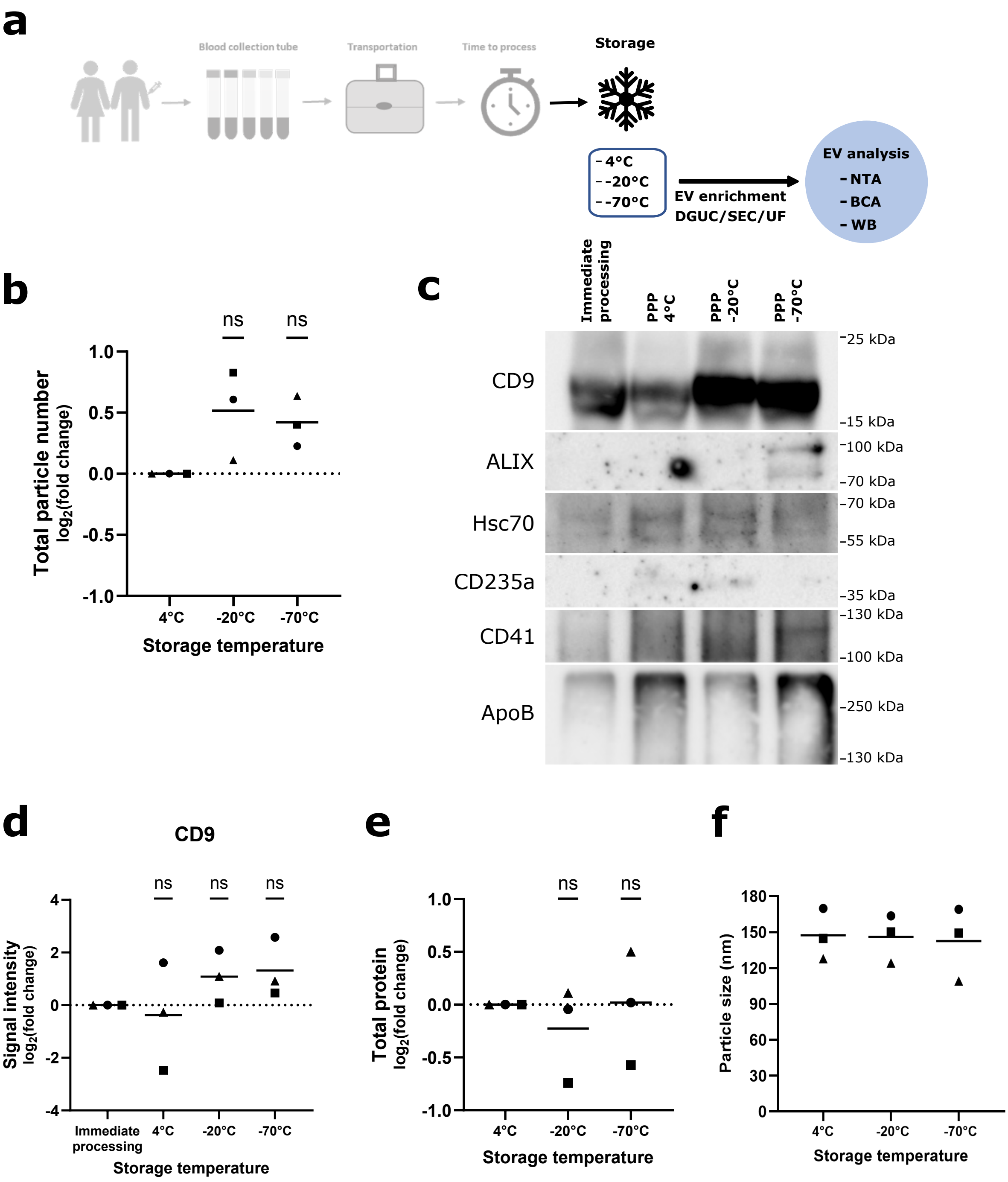

Supplementary Figure S4

Supplementary Figure S4: Storage temperature of platelet-poor plasma

a. A set of three peripheral blood samples were collected from n = 3 healthy volunteers in a Streck Cell-Free DNA BCT. Platelet poor plasma (PPP) was stored for two weeks at 4°C, -20°C or -70°C. EV-enrichment was performed by DGUC, followed by SEC and then UF. EV-enriched fractions were analyzed by nanoparticle tracking analysis (NTA), bicinchoninic acid assay (BCA) and western blot (WB)

b. Particle numbers measured by NTA. Data normalized to storage at 4°C. Mean of normalized value is displayed for every temperature and statistical significance is assessed using a one sample t test with p=0.1353 (-20°C) and p=0.0716 (-70°C).

c. Representative immunoblotting from particles purified after the different storage conditions tested. Original blots are presented in Supplementary Figure S13

d. Quantification of the immunoblot signal for CD9 of n=3 independent experiments. Data normalized to immediate processing (no storage). Mean of normalized value is displayed for every temperature and statistical significance is assessed using a one sample t test with p=0.7783 (4°C), p=0.2001(-20°C) and p=0.1764 (-70°C).

e. Total protein obtained from each storage condition after lysing EV-enriched fractions, measured by BCA. Data normalized to storage at 4°C. Mean of normalized value is displayed for every temperature and statistical significance is assessed using a one sample t test with p=0.4801 (-20°C) and p=0.9613 (-70°C).

f. Median particle size obtained after thawing the PPP samples and EV-enrichment. Mean value is displayed for every temperature.

Data in b, d-f is derived from n=3 biological replicates. Symbols signify independent biologial replicates.

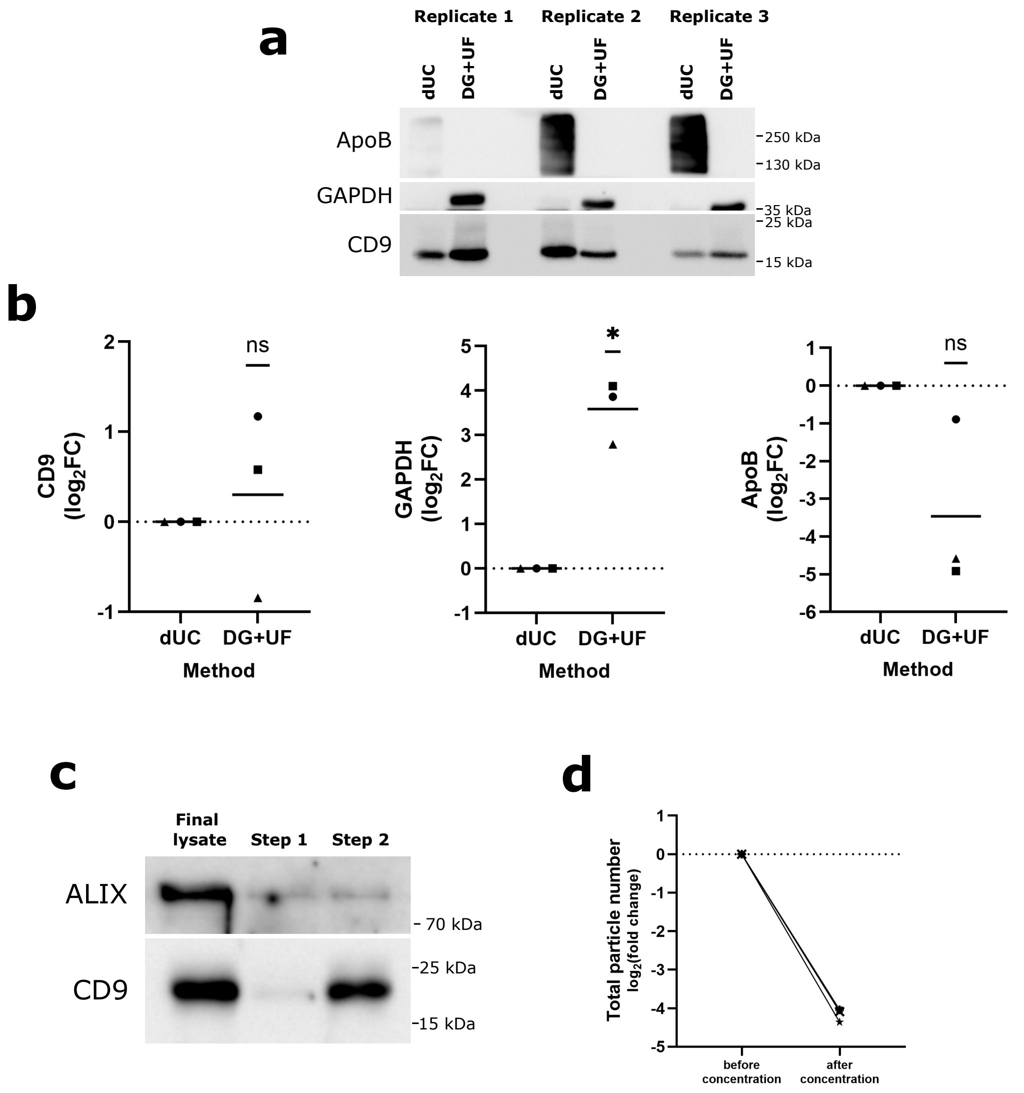

Supplementary Figure S5

Supplementary Figure S5: Choice of EV isolation method

a. Immunoblot and b. quantification of EV-derived protein markers purified in n=3 biological replicates either by differential ultracentrifugation or density gradient ultracentrifugation. Data normalized to dUC condition. Mean of normalized value is displayed for each time condition and statistical significance is assessed using one sample t test with $p=0.6635$ (CD9), *$p= 0.0124$ (GAPDH) and $p=0.115$ (ApoB).

dUC = differential ultracentrifugation + Size exclusion chromatography, DG+UF = density gradient ultracentrifugation + size exclusion chromatography + ultrafiltration.

c. Immunoblot to assess the EV loss throughout the process of isolation by density gradient and size exclusion chromatography.

Final lysate = lysed EVs after removal from Amicon filter following DG and SEC.

Step 1 = EV-enriched volume from the density gradient was collected in an 1.5 mL eppi tube. After removal of this volume, lysis buffer was added to the tube to assess remaining particles.

Step 2 = EV-enriched fractions from SEC were collected directly in an Amicon ultrafiltration unit. After concentrating the sample, the concentrate was removed and lysis buffer was added to the filter to assess remaining particles.

d. Calculated total particle number based on NTA measurement before and after ultrafiltration of EV-enriched fractions.

Data in b and d is derived from n=3 biological replicates. Symbols signify independent biologial replicates. Symbols in b correspond with Figure 5.

Original blots for a. and c. are presented in Supplementary Figure S14.

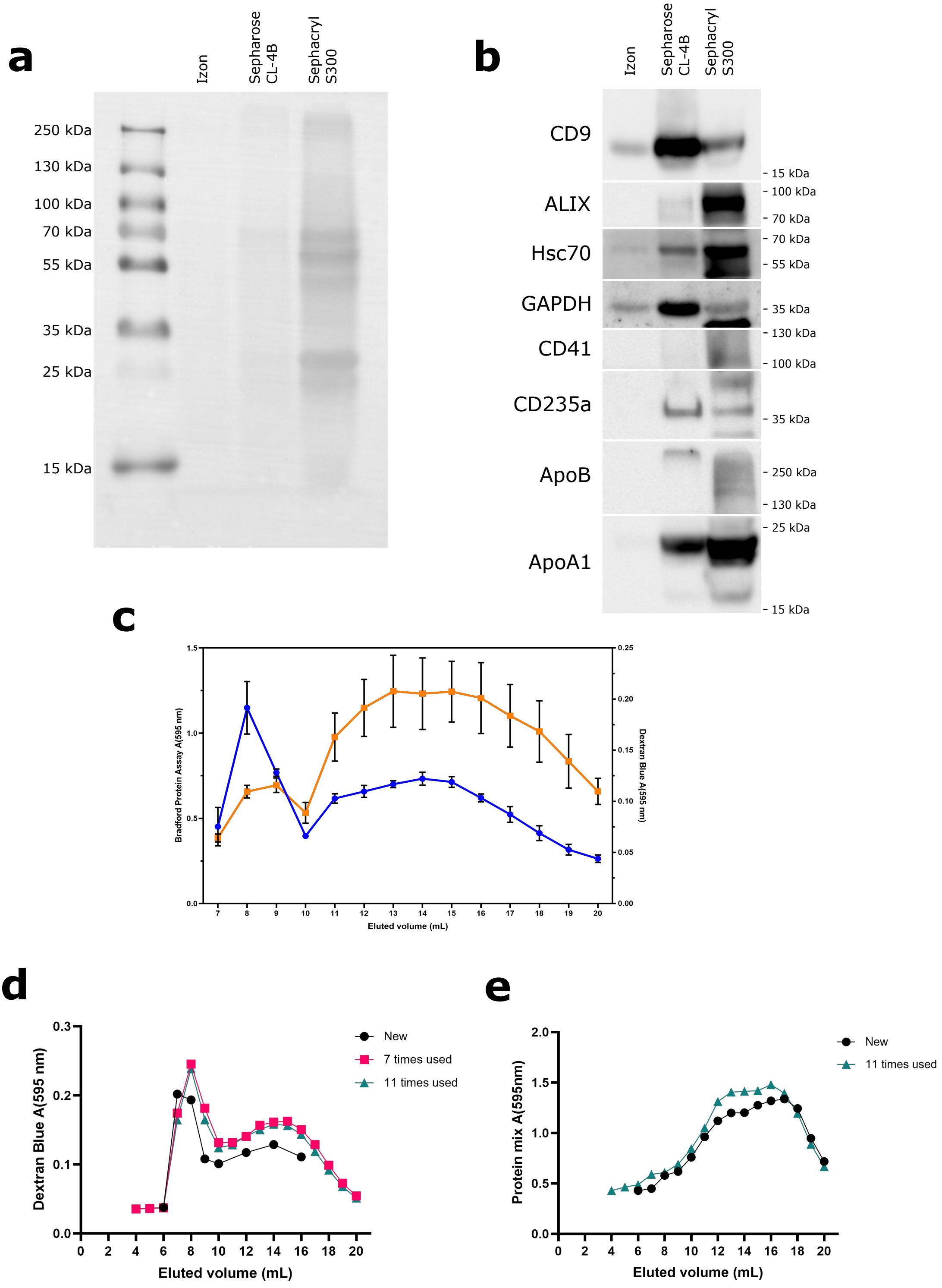

Supplementary Figure S6

Supplementary Figure S6: Size exclusion columns

a. Representative Ponceau colorimetric and b. immunoblotting image of the pooled EV-enriched fractions obtained after size exclusion chromatography performed with the different resins.

Original blots are presented in Supplementary figure S15.

c. Assessment of Sepharose CL-4B elution profiles using as representative standards Dextran Blue (2g/L)(right y axis) and protein mix of Thyroglobulin (2g/L) and bovine serum albumin (BSA, 4g/L) (left y axis) as quality control to evaluate reproducibility (n=6 biological replicates). Mean and standard deviation are displayed for every eluted volume point.

d. Elution profiles of Dextran Blue (2g/L) obtained from the same Sepharose CL-4B column freshly prepared, after 7 times or after 11 times usage, respectively.

e. Elution profiles of protein mix (Thyroglobulin (2g/L) + BSA (4g/L)) obtained from the same Sepharose CL-4B as in d, freshly prepared or after 11 times usage.

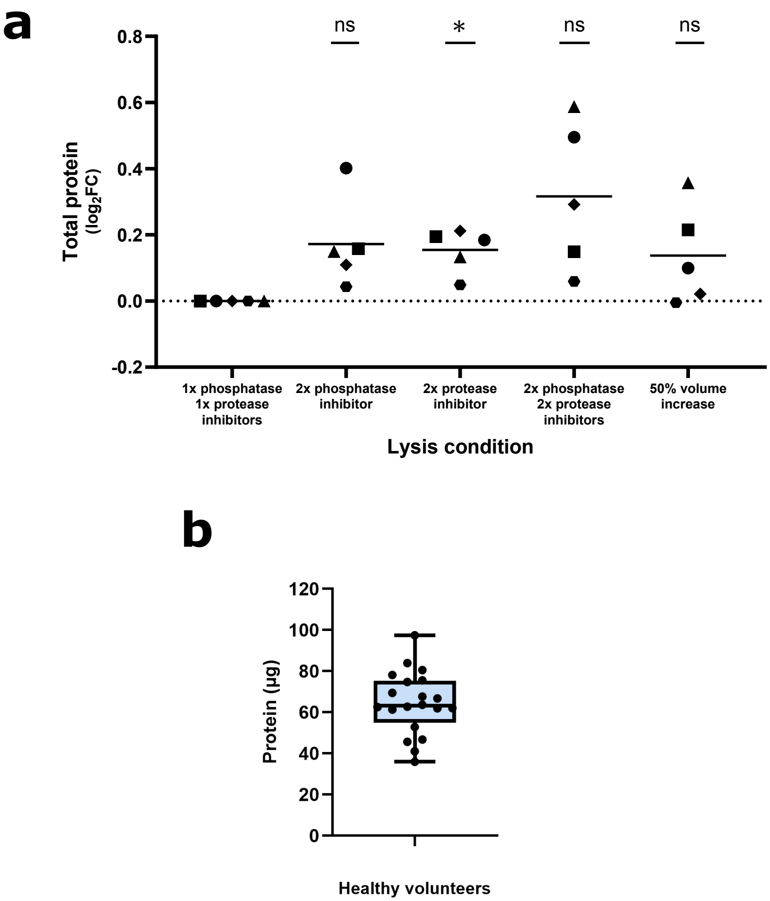

Supplementary Figure S7

Supplementary Figure S7: Lysis buffer and protein yield

a. EV-enriched SEC fractions were lysed with RIPA buffer and the impact of different concentrations of phosphatase and protease inhibitors on protein recovery was measured by BCA (n=5 biological replicates). Data normalized to the 1xphosphatase 1xprotease inh. condition. Mean of normalized value is displayed for each lysis condition and statistical significance is assessed using one sample t test (Bonferroni-Holm-adjusted p-values) with p=0.1023 (2x phosphatase inh.), *p=0.0252 (2x protease inh.), p=0.1023 (2x phosphatase 2x protease inh.) and p=0.1090 (50% volume inccrease). Symbols signify independent biological replicates.

b. Protein yields obtained from blood donor healthy volunteers after EV enrichment by DGUC, SEC and UF (n=20 biological replicates).

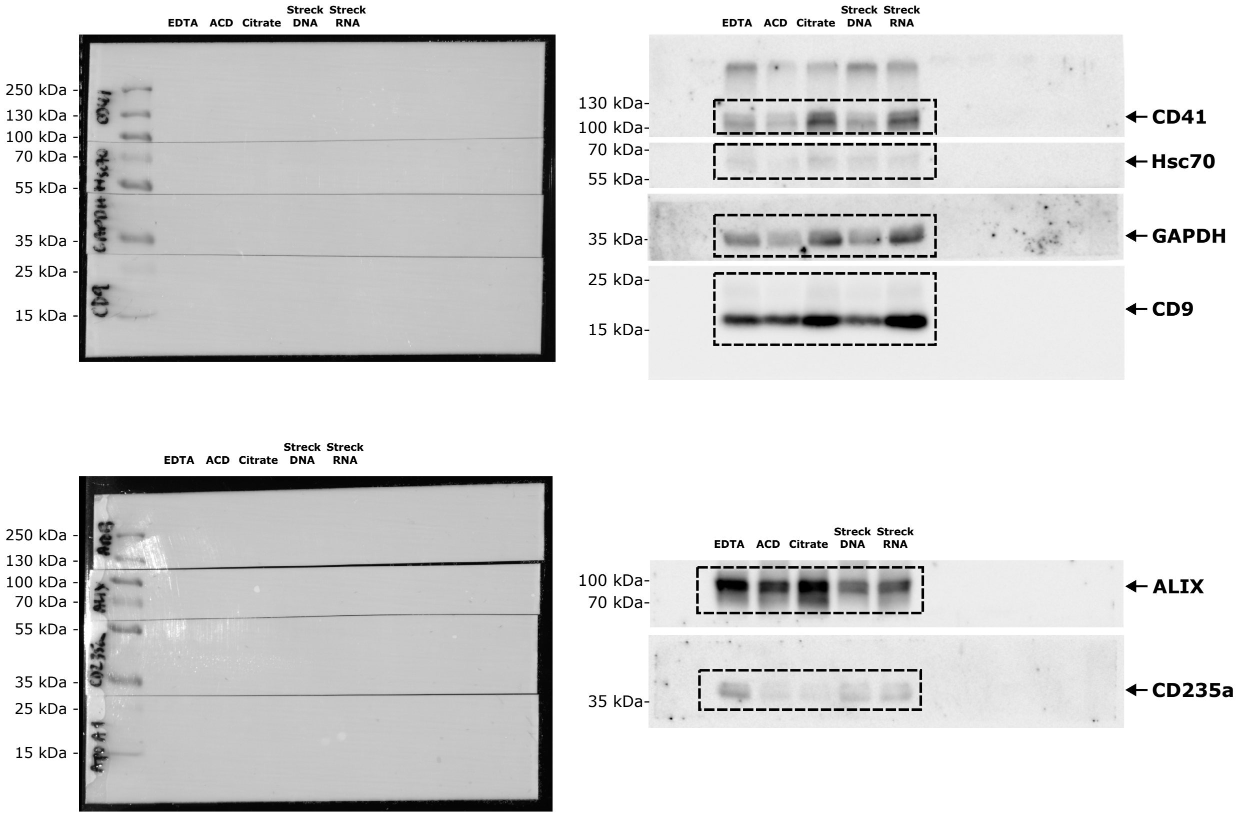

Supplementary Figure S8

Supplementary Figure S8: Original Immunoblots from Figure 2
Original colorimetric and chemiluminescence images of the Immunoblot displayed in Figure 2. Before incubation with the primary antibody, the membrane was divided to be incubated with the corresponding antibody according its molecular weight. The grouping of the images correspond to one membrane used to test different proteins within the same sample. The chemiluminescent images on the right correspod to the colorimetric images on the left.

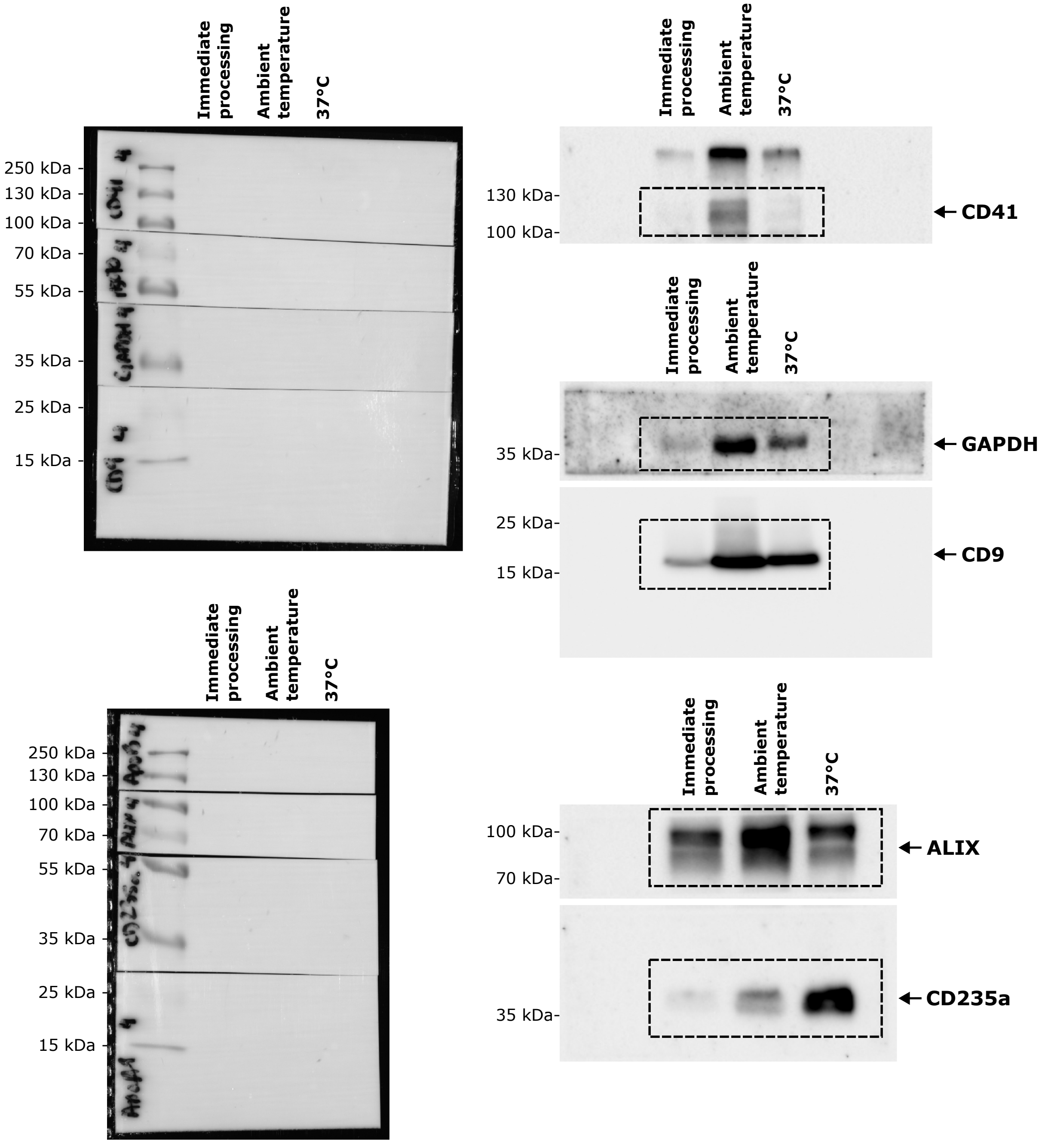

Supplementary Figure S9

Supplementary Figure S9: Original Immunoblots from Figure 3

Original colorimetric and chemiluminescence images of the Immunoblot displayed in Figure 3. Before incubation with the primary antibody, the membrane was divided to be incubated with the corresponding antibody according its molecular weight. The grouping of the images correspond to one membrane used to test different proteins within the same sample. The chemiluminescent images on the right correspod to the colorimetric images on the left.

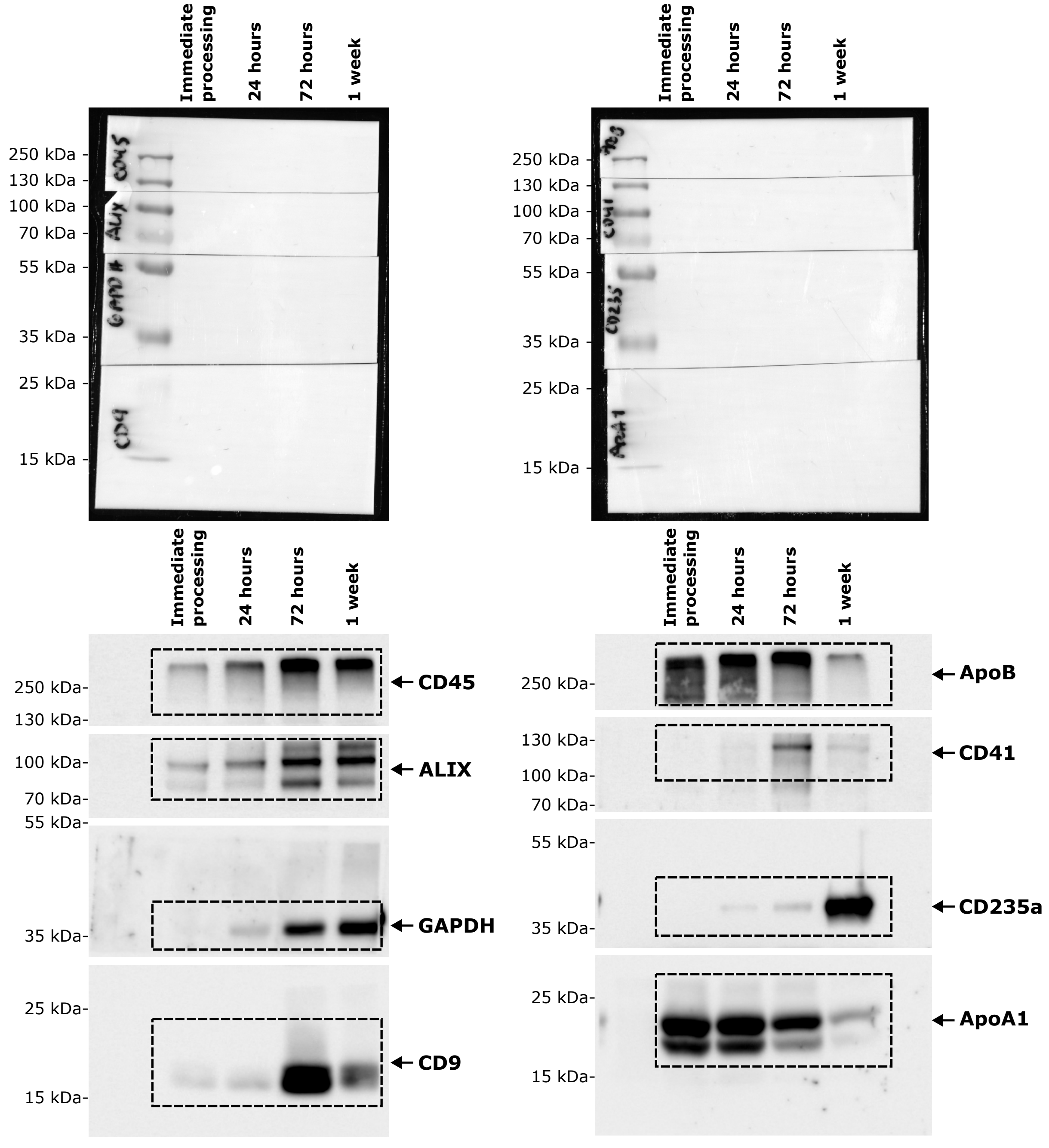

Supplementary Figure S10

Supplementary Figure S10: Original Immunoblots from Figure 4

Original colorimetric and chemiluminescence images of the Immunoblot displayed in Figure 4. Before incubation with the primary antibody, the membrane was divided to be incubated with the corresponding antibody according its molecular weight. The grouping of the images correspond to one membrane used to test different proteins within the same sample. The chemiluminescent images on the bottom correspod to the colorimetric images on the top

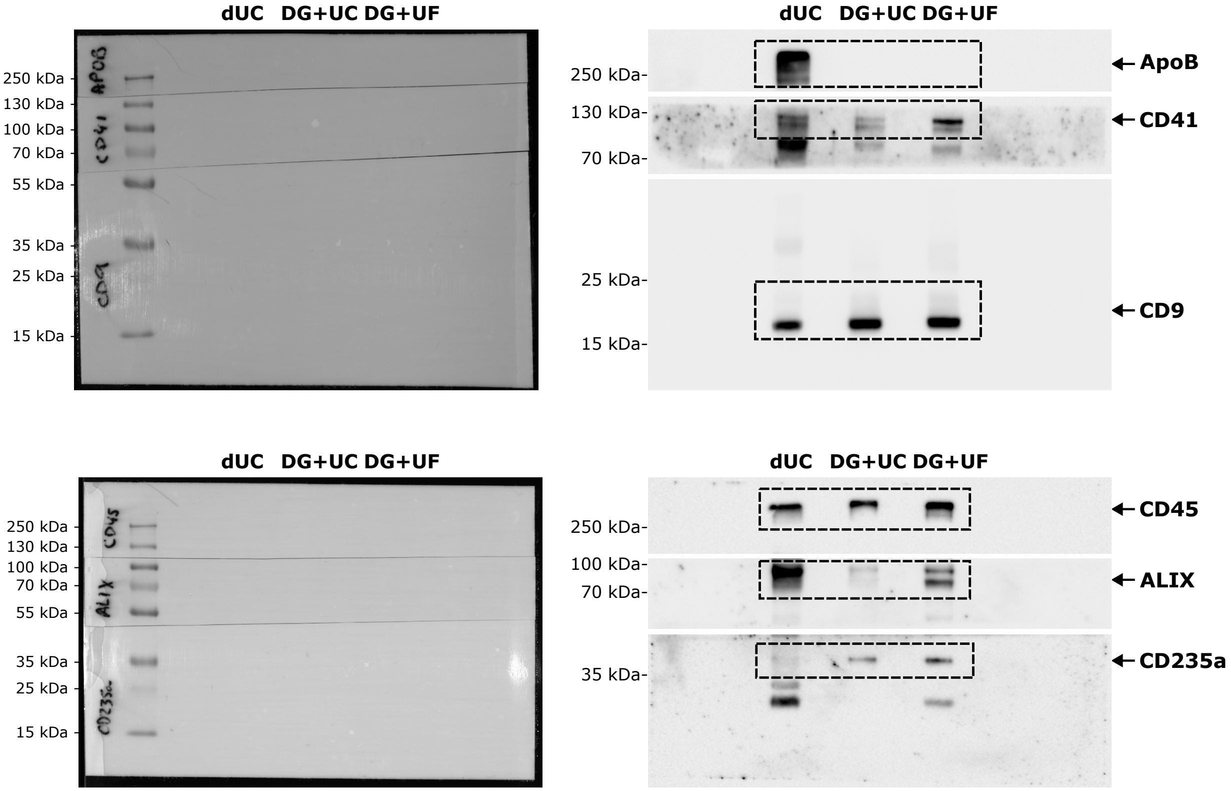

Supplementary Figure S11

Supplementary Figure S11: Original Immunoblots from Figure 5

Original colorimetric and chemiluminescence images of the Immunoblot displayed in Figure 5. Before incubation with the primary antibody, the membrane was divided to be incubated with the corresponding antibody according its molecular weight. The grouping of the images correspond to one membrane used to test different proteins within the same sample. The chemiluminescent images on the right correspod to the colorimetric images on the left.

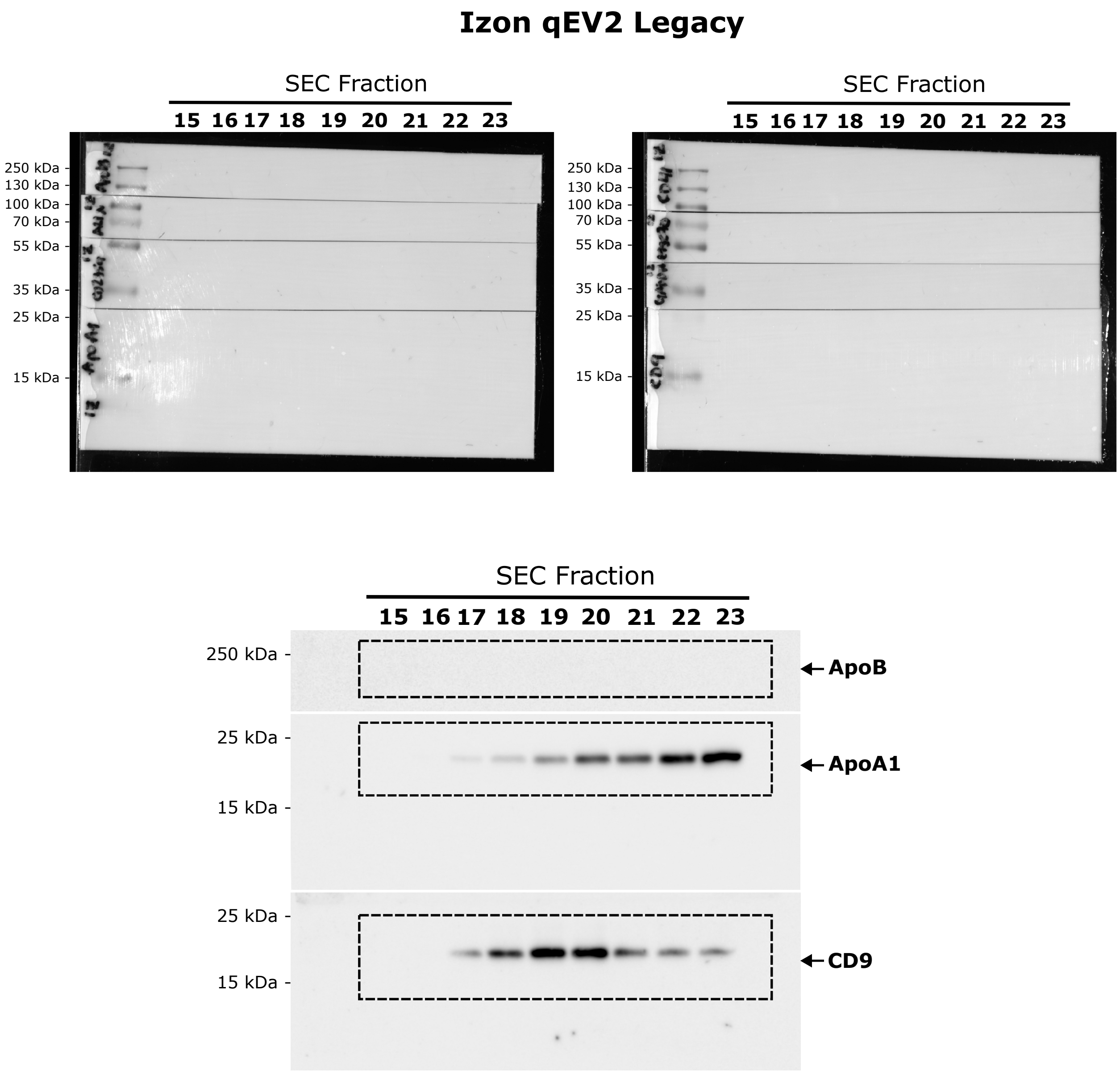

Supplementary Figure S12

Sepharose CL-4B

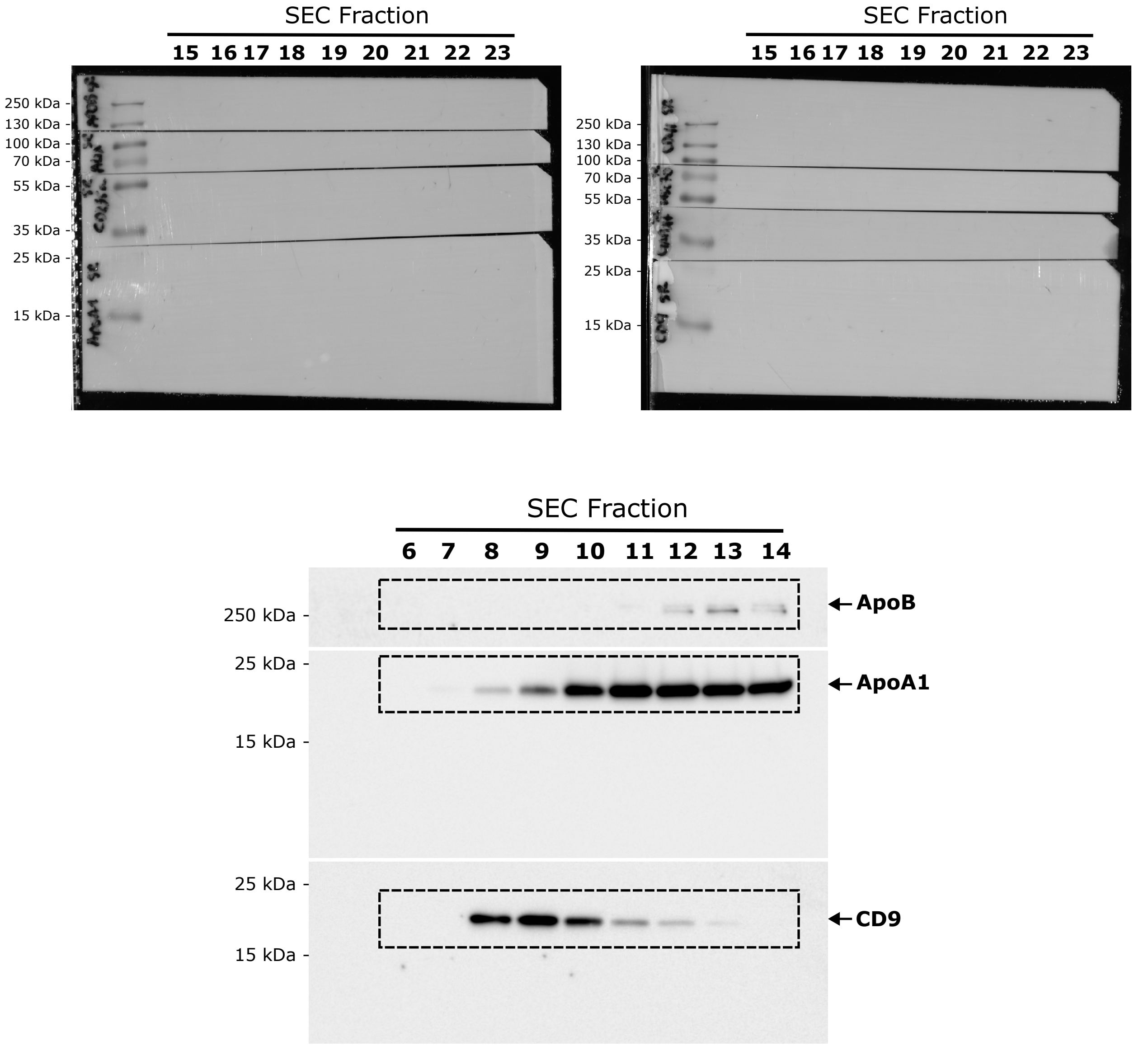

Supplementary Figure S12 (continued)

Sephacryl S300

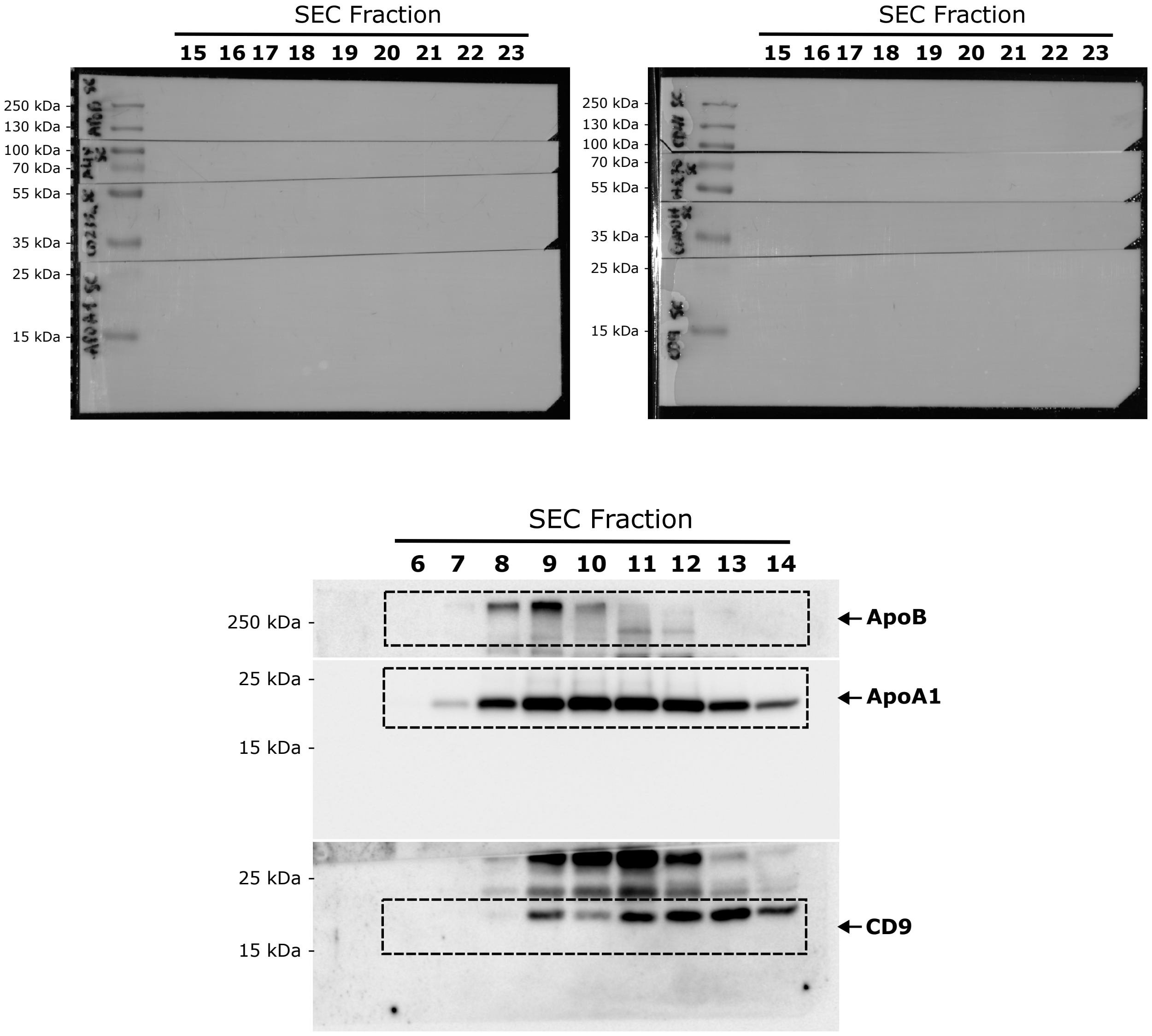

Supplementary Figure S12 (continued)

Supplementary Figure S12: Original Immunoblots from Figure 6
Original colorimetric and chemiluminescence images of the Immunoblot displayed in Figure 6. Before incubation with the primary antibody, the membrane was divided to be incubated with the corresponding antibody according its molecular weight. The grouping of the images correspond to one membrane used to test different proteins within the same sample. The chemiluminescent images on the bottom correspod to the colorimetric images on the top.

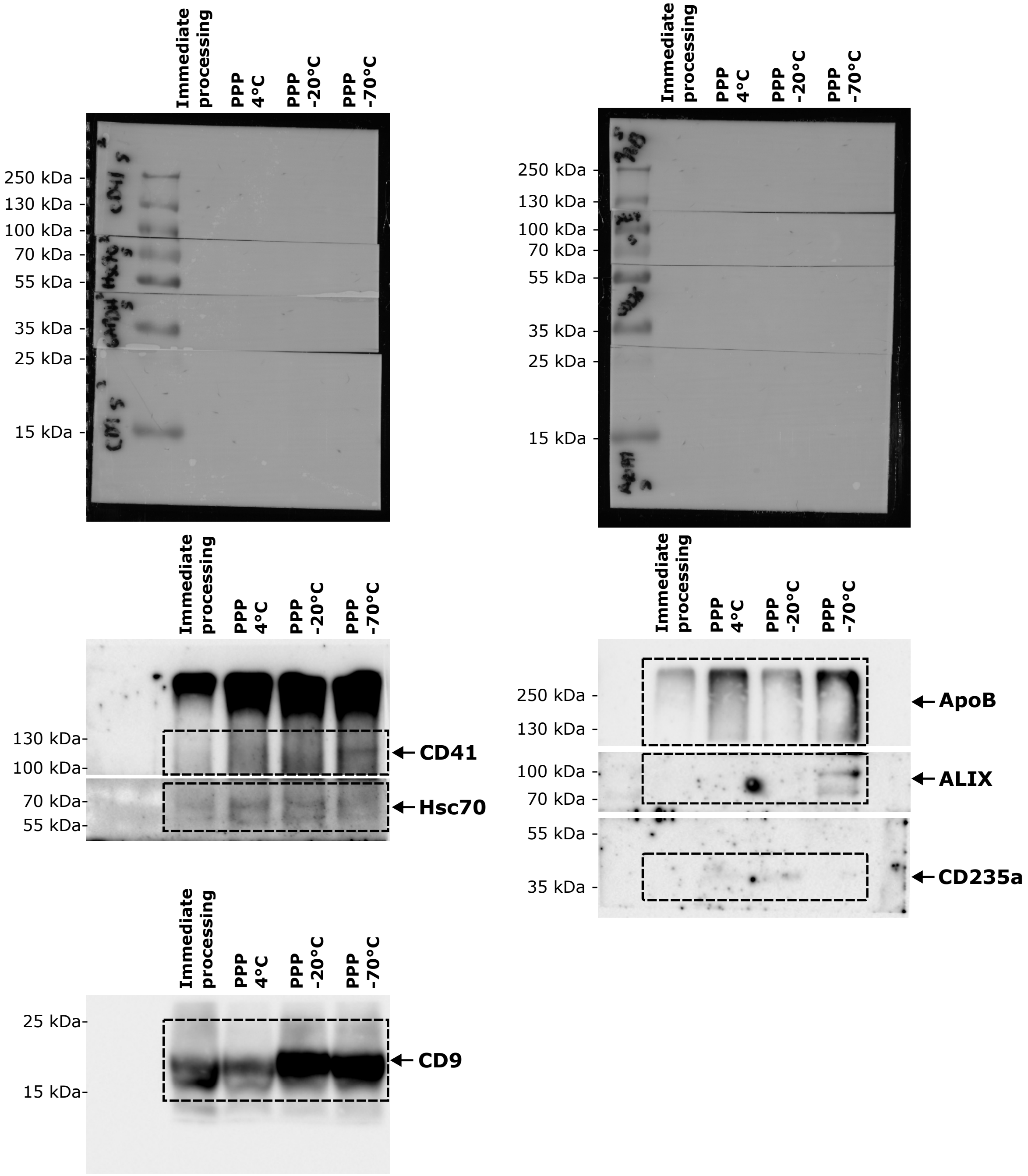

Supplementary Figure S13

Supplementary Figure S13: Original Immunoblots from Figure S4

Original colorimetric and chemiluminescence images of the Immunoblot displayed in Figure S4. Before incubation with the primary antibody, the membrane was divided to be incubated with the corresponding antibody according its molecular weight. The grouping of the images correspond to one membrane used to test different proteins within the same sample. The chemiluminescent images on the bottom correspod to the colorimetric images on the top.

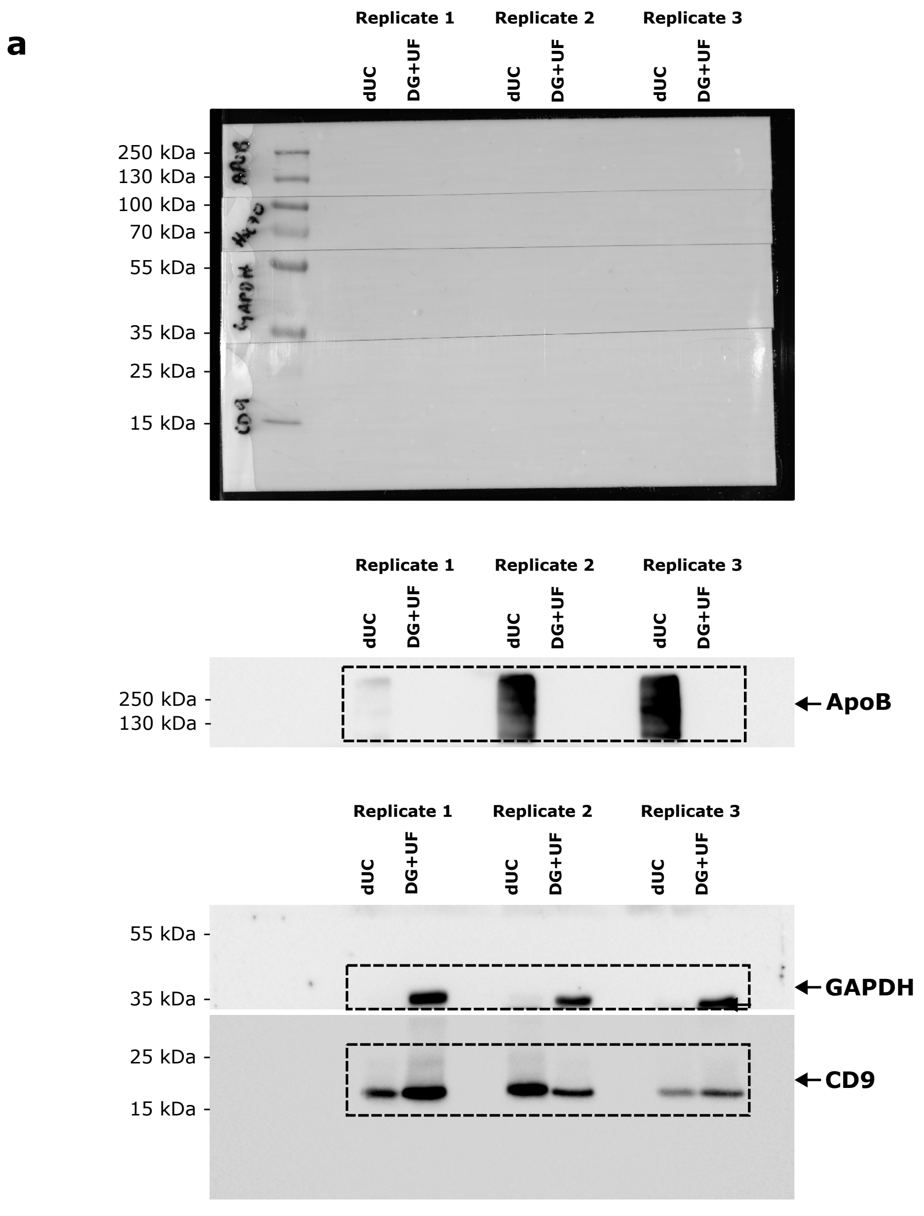

Supplementary Figure S14

b

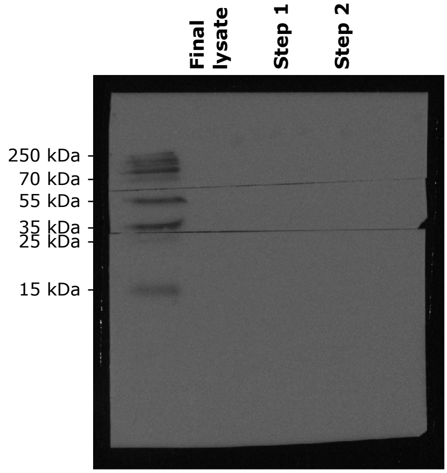

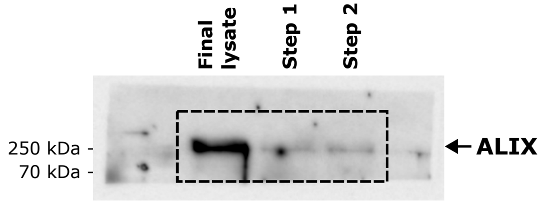

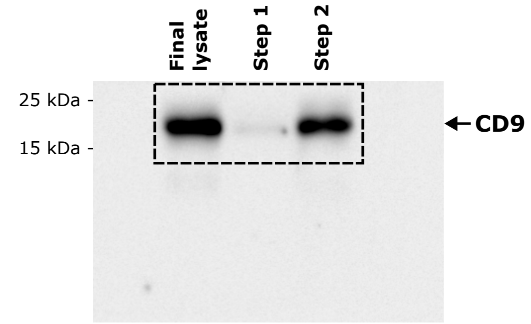

Supplementary Figure S14 (continued)

Supplementary Figure S14: Original Immunoblots from Figure S5

Original colorimetric and chemiluminescence images of the Immunoblot displayed in Figure S5a (a) and S5c (b). Before incubation with the primary antibody, the membrane was divided to be incubated with the corresponding antibody according its molecular weight. The grouping of the images correspond to one membrane used to test different proteins within the same sample. The chemiluminescent images on the bottom correspod to the colorimetric images on the top.

a

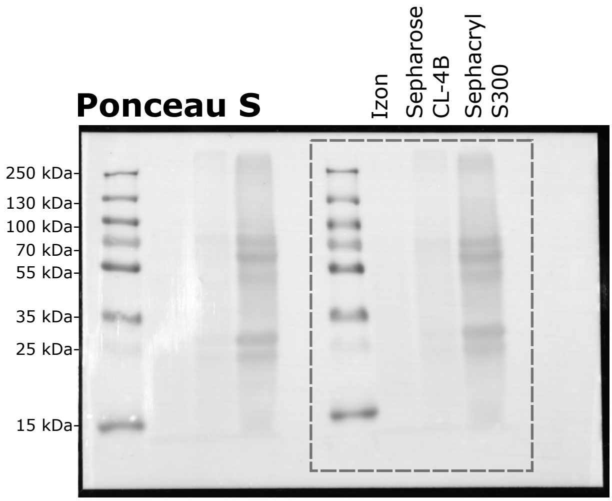

b

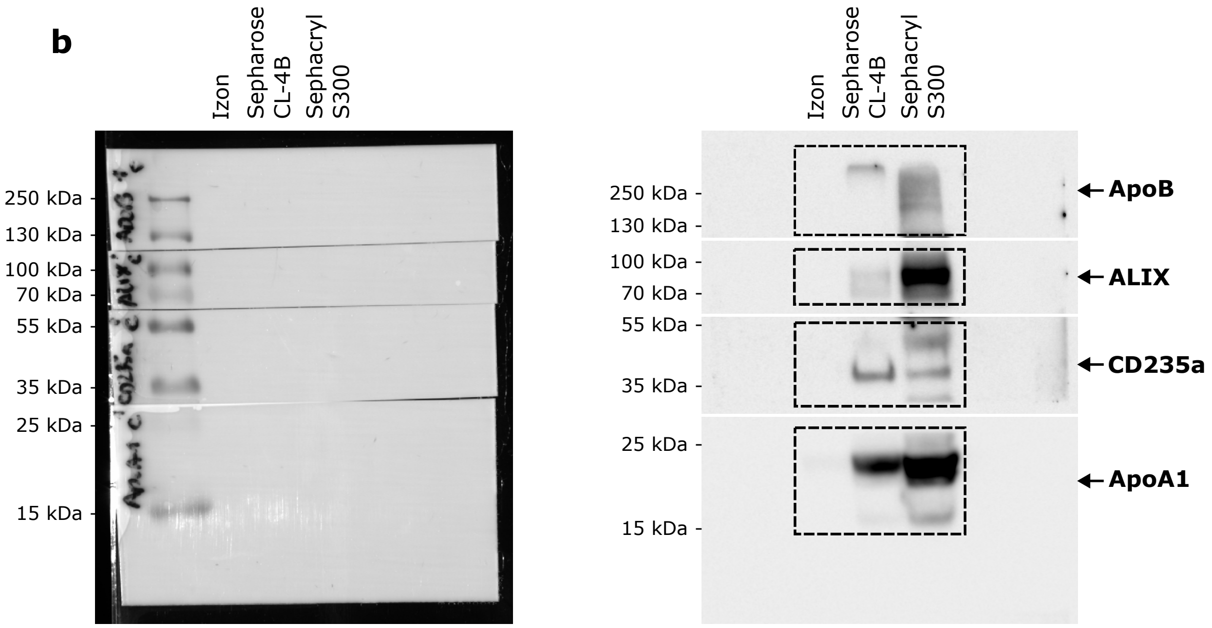

Supplementary Figure S15

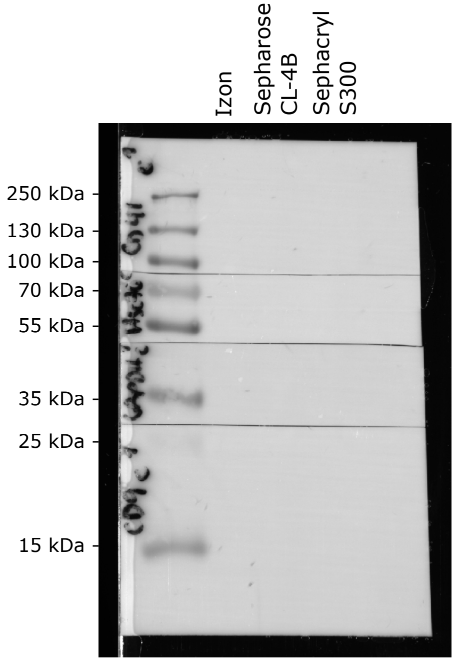

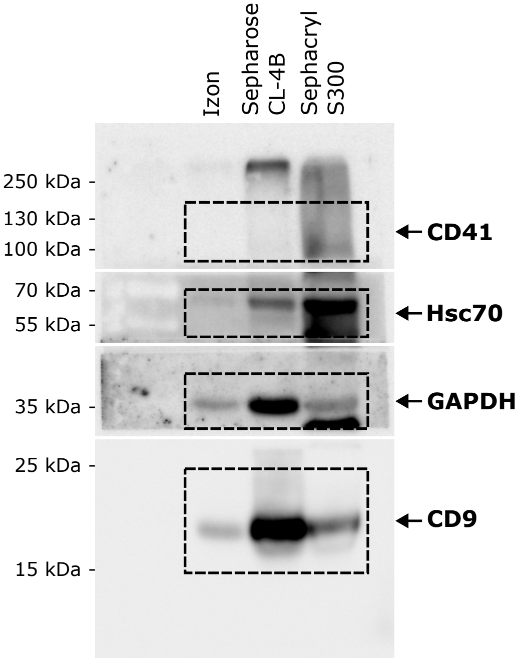

Supplementary Figure S15 (continued)

Supplementary Figure S15: Original Immunoblots from Figure S6
Original colorimetric and chemiluminescence images of the Immunoblot displayed in Figure S6a (a) and S5b(b). Before incubation with the primary antibody, the membrane was divided to be incubated with the corresponding antibody according its molecular weight. The grouping of the images correspond to one membrane used to test different proteins within the same sample. In b, the chemiluminescent images on the right correspod to the colorimetric images on the left.
